# Diversification of the plant-specific hybrid glycine-rich protein (HyGRP) genes in cereals

**DOI:** 10.3389/fpls.2014.00489

**Published:** 2014-09-24

**Authors:** Kenji Fujino, Mari Obara, Koji Sato

**Affiliations:** NARO Hokkaido Agricultural Research Center, National Agricultural Research OrganizationSapporo, Japan

**Keywords:** gene family, HyP/GRPs, orthologue, *Oryza sativa* L., rice

## Abstract

Plant-specific hybrid proline- or glycine-rich proteins (HyP/GRPs) are involved in diverse gene functions including plant development and responses to biotic and abiotic stresses. The quantitative trait locus, *qLTG3-1*, enhances seed germination in rice under low-temperature conditions and encodes a member with a glycine-rich motif of the HyP/GRP family. The function of this gene may be related to the weakening of tissue covering the embryo during seed germination. In the present study, the diversification of the HyP/GRP gene family was elucidated in rice based on phylogenetic relationships and gene expression levels. At least 21 members of the HyP/GRP family have been identified in the rice genome and clustered in five regions on four chromosomes by tandem and chromosomal duplications. Of these, *OsHyPRP05* (*qLTG3-1*) and its paralogous gene, *OsHyPRP21*, had a glycine-rich motif. Furthermore, orthologous genes with a glycine-rich motif and the HyP/GRP gene family were detected in four genome-sequenced monocots: 12 in barley, 10 in *Brachypodium*, 20 in maize, and 28 in sorghum, using a BLAST search of *qLTG3-1* as the query. All members of the HyP/GRP family in these five species were classified into seven main groups, which were clustered together in these species. These results suggested that the HyP/GRP gene family was formed in the ancestral genome before the divergence of these species. The collinearity of chromosomal regions around *qLTG3-1* and its orthologous genes were conserved among rice, *Brachypodium*, sorghum, and maize, indicating that *qLTG3-1* and orthologous genes conserve gene function during seed germination.

## Introduction

Gene duplication contributes to genetic complexity during evolution (Hughes, 1994; Lynch and Force, 2000; Gu et al., 2003). One of the duplicated genes becomes a pseudogene through the accumulation of deleterious mutations, or duplicated genes adopt a subset of functions from the ancestral gene. Extant plant genomes may all result from whole genome duplication and diploidization (Adams and Wendel, 2005). Major losses, structural and functional divergence, or concerted evolution have been observed in eukaryote genomes via their whole genome duplication (Ahn and Tanksley, 1993; Wang et al., 2005; Scannell et al., 2006; Sjödin et al., 2008). The availability of the genome sequences of major cereals, rice (International Rice Genome Sequencing Project, 2005), sorghum (Paterson et al., 2009), barley (The International Barley Genome Sequencing Consortium, 2012), and maize (Schnable et al., 2009), has provided an opportunity for whole genome annotations and comparative genomic research to understand functional diversity among gene families.

Hybrid proline- or glycine-rich proteins (HyP/GRP) are plant-specific and putative cell-wall/plasma membrane-associated proteins. These mature proteins have two distinct domains: a hydrophilic proline-rich or glycine-rich repetitive domain (PRD or GRD, respectively) at the N-terminus, and a hydrophobic domain with eight cysteine residues in a specific order called the eight-cysteine motif (8CM) at the C-terminus. Although the HyP/GRP family has a unique structural feature, their proposed molecular functions vary and include plant development (Wu et al., 1993; Holk et al., 2002; Blanco-Portales et al., 2004), responses to various stresses including cold/heat, drought, and salinity (Deutch and Winicov, 1995; Goodwin et al., 1996; Zhang and Schläppi, 2007; Fujino et al., 2008b; Priyanka et al., 2010; Tan et al., 2013), and defenses against pathogens (Josè-Estanyol et al., 1992; He et al., 2002; Bouton et al., 2005; Weyman et al., 2006; Jung et al., 2009; Yeom et al., 2012). HyP/GRPs form a multi-gene family in plant species and have different gene expression profiles (Dvoráková et al., 2007). Therefore, little is known about their molecular functions and diversifications in plant development and responses to biotic and abiotic stresses.

Rice, as one of the most important cereals in the world, has become the most important model cereal for functional genomics. In many Asian countries, the direct seeding method has become increasingly important (Dingkuhn et al., 1992). Therefore, low temperature during the sowing at high altitudes and latitudes delays emergence of the rice seedling from water (Peterson et al., 1978), causing serious decreases of yields. We previously identified a quantitative trait locus (QTL) for low temperature tolerance at the seed germination stage, *qLTG3-1* (Fujino et al., 2004, 2008b). This gene encodes one member with a glycine-rich motif of the HyP/GRP gene family in the rice genome. Although the molecular function of *qLTG3-1* remains unknown, histological analyses indicated that the tissue-specific expression of *qLTG3-1* is closely associated with the vacuolation of cells in the tissues covering the embryo. Based on these findings, *qLTG3-1* was considered to be involved in tissue weakening. Genome-wide expression analysis demonstrated that genes involved in defense responses were up-regulated by *qLTG3-1* (Fujino and Matsuda, 2010). These findings indicated that the expression of *qLTG3-1* was necessary for the expression of defense response genes in low-temperature germinability in rice.

Genes that are highly similar to *qLTG3-1* have been shown to exist in the genomes of plants other than rice (Fujino et al., 2008b). Thus, it remains unclear whether these genes have the same function as *qLTG3-1*. In the present study, the phylogenetic relationships between and gene expression profiles of HyP/GRPs were characterized in rice to clarify whether other members had redundant or novel functions. Genome sequencing enabled us to perform comprehensive surveys of orthologous gene families across species (Hamilton and Buell, 2012). Comparative genomic analysis of the chromosomal regions around the *qLTG3-1* orthologous genes in monocots including rice (International Rice Genome Sequencing Project, 2005), sorghum (Paterson et al., 2009), barley (The International Barley Genome Sequencing Consortium, 2012), maize (Schnable et al., 2009), and *Brachypodium* (The International Brachypodium Initiative, 2010) strongly suggested that *qLTG3-1* orthologous genes with a glycine-rich motif have conserved gene function.

## Materials and methods

### Plant materials

The rice varieties Hokkaiwase, Kitaake, and Hoshinoyume were used for the gene expression analysis. The genotypes of *qLTG3-1* in Hokkaiwase, Kitaake, and Hoshinoyume were the wild type, single amino acid substitute type, and loss-of-function type, respectively (Fujino and Iwata, 2011; Fujino and Sekiguchi, 2011). These varieties were cultivated in an experimental paddy field at Hokkaido Agricultural Research Center, Sapporo, Japan, 43°00′N latitude, in 2013. Seeds were harvested at the maturing stage and were then maintained at room temperature. These seeds were used in experiments 4 months after harvesting. Seeds were incubated at 15 and 30°C in dark conditions (Fujino et al., 2004). After being incubated, seed samples from each time point were frozen immediately in liquid nitrogen and stored at −80°C for RNA extraction.

In the analysis of the sequence diversity of *Os10g0554800*, a paralogous gene of *qLTG3-1*, a set of 58 genetically diverse varieties was used, which represents the wide genetic diversity among cultivated rice varieties termed world rice core collection (WRC) (Kojima et al., 2005). Seeds were provided by the Local Independent Administrative Agency Hokkaido Research Organization and National Institute of Agrobiological Sciences, Japan.

### Database search for HyP/GRP genes

To identify the HyP/GRP gene family in the rice genome, we employed BlastP searches of RAP-DB (http://rapdb.dna.affrc.go.jp/) and MSU-RGAP (http://rice.plantbiology.msu.edu/) using the protein sequence of *qLTG3-1* as a query. The HyP/GRP gene family in monocots was searched by BlastP programs. The protein sequences in maize, sorghum, and *Brachypodium* were retrieved from Phytozome version 10 (http://phytozome.jgi.doe.gov/pz/portal.html). Barley gene sequences were retrieved from the IPT Barley blast server (http://webblast.ipk-gatersleben.de/barley/). The 1 kb upstream region from the transcription start site on each gene was also retrieved from databases as the promoter sequence.

Multiple sequence alignment of the HyP/GRP protein sequences was performed using the ClustalW method with MEGA version 6 built in with a default setting (http://www.megasoftware.net/). A phylogenic tree was constructed by the neighbor-joining method considering 1000 replications with bootstrap analyses. The similarity of the amino acid sequences and identities of the promoter sequences among the HyP/GRP gene family were calculated using alignment data by the SIAS program with a default setting (http://imed.med.ucm.es/Tools/sias.html). The *cis*-acting elements in the promoter region were analyzed using the MEME suite (http://meme.nbcr.net/meme/) with the following parameters. The optimum width of each motif was between 6 and 20 bp. The number of differential motifs was 20, while that of the minimum motif site was 5. *E* = 1.1e^+4^ was used as a threshold for shuffle sequences.

### Comparative analysis of genome structures around *qLTG3-1* orthologous genes

Syntenic dotplots for the chromosomal regions around *qLTG3-1* orthologous genes were generated using PipMaker (http://pipmaker.bx.psu.edu/pipmaker/). The orthologous genomic regions were identified through comparative genomics analysis of the putative highly conserved gene pairs in monocots. The 163 kb in rice chromosome 3 including 22 genes, the 117 kb in Brachypodium chromosome 1 including 21 genes, the 200 kb in sorghum chromosome 1 including 15 genes, the 383 kb in maize chromosome 1 including 17 genes, and the 209 kb in barley chromosome 4 including 12 genes were used for the initial analysis. The CDSs in these orthologous genomic regions were then used to analyze collinearity among the monocots.

### DNA analysis

Total DNA was isolated from young leaves using the CTAB method (Murray and Thompson, 1980). PCR, electrophoresis, and sequencing were performed as described previously (Fujino et al., 2004, 2005, 2010). The 1128-bp *Os10g0554800* region was sequenced, including the 364-bp 5′ upstream region, 504-bp coding region, and 260-bp 3′ downstream region. PCR products were sequenced directly using cycle sequencing with BigDye terminators (Applied Biosystems) on a Prism 3700 automated sequencer (Applied Biosystems). The sequences of four *Os10g0554800* alleles were deposited in GenBank as Accession Nos. AB973302–AB973304.

### RNA analysis

RNA extraction and semi-quantitative RT-PCR analysis were performed as described previously (Fujino et al., 2008a,b; Fujino and Matsuda, 2010). Total RNA was extracted from 10 embryos of seeds during seed germination because *qLTG3-1* was specifically expressed in the embryos of germinating seeds (Fujino et al., 2008b). In addition, total RNA from the roots of 4-day-old seedlings and the 3rd leaf blades of 3-week-old seedlings were used. In semi-quantitative RT-PCR, each PCR reaction (10 μl) contained 1 μl of a five-fold-diluted cDNA template. The gene-specific primer sets and PCR conditions are listed in Supplemental Table S1. PCR was performed under the same conditions as those for RT-PCR using RNA without reverse transcription to determine contamination with genomic DNA. To validate the results obtained, each PCR experiment was repeated three times.

## Results and discussion

### HyP/GRP gene family in rice

**Figure 1:**
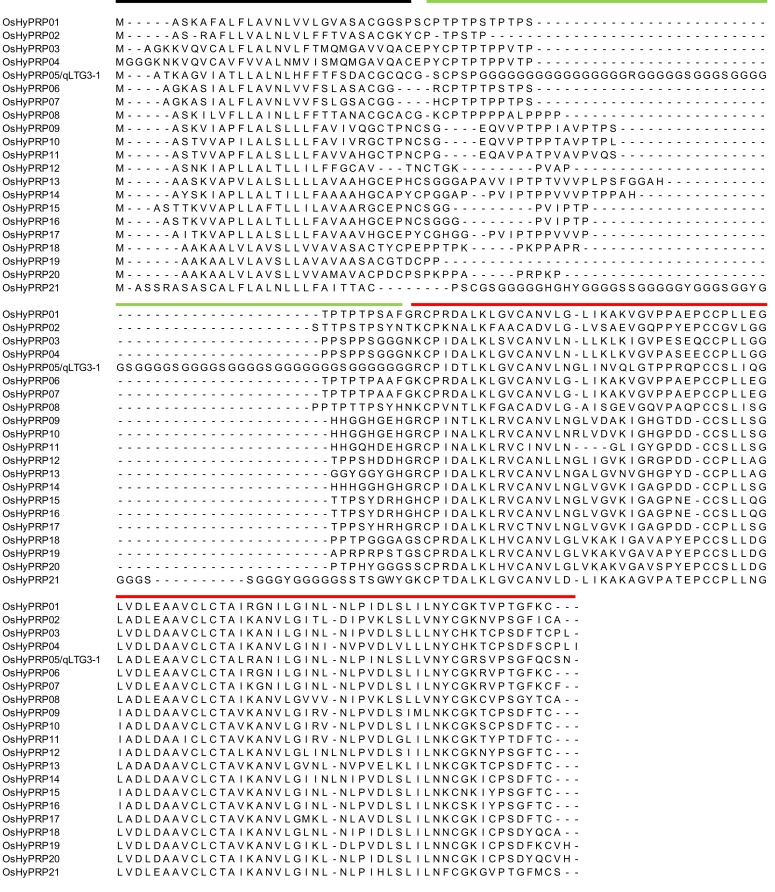
**Sequence alignment of the HyP/GRP gene family in rice**. Black, green, and red lines show three conserved regions: an N-terminal region (region A), variable P/GRD region (region B), and conserved 8CM region (region C), respectively.

To identify members of the HyP/GRP gene family in rice, the reference Nipponbare genome sequence was searched using the *qLTG3*-1 proteins as queries. A total of 21 genes were identified as putative HyP/GRP genes (Table 1). Amino acid alignments ranged between 124 and 184 amino acids. We renamed them *OsHyPRP01* to *OsHyPRP21* based on their order on the chromosome. *OsHyPRP05* was *qLTG3-1*, which controlled low-temperature tolerance at the seed germination stage (Fujino et al., 2004, 2008b). *OsHyPRP01* and *OsHyPRP13* were *RCc3* and *RCc2*, respectively, which were root-specific proteins (Xu et al., 1995). The amino acid alignments of OsHyPRP revealed three conserved regions: an N-terminal region (region A), variable P/GRD region (region B), and conserved 8CM region (region C) (Figure 1). All genes, except for *OsHyPRP21*, were clustered in five regions spanning 9573–38,960 bp intervals on four chromosomes. *OsHyPRP21* was located on chromosome 10 apart from the 89,260 bp of *OsHyPRP20*. Clusters derived by tandem duplication on chromosomes 2 and 4 and chromosomes 3 and 10 were paralogous chromosomal regions. These two pairs were previously shown to be involved in 10 major chromosome-to-chromosome duplication relationships in the rice genome (Throude et al., 2009).

**Table 1:** **The HyP/GRP gene family in rice**.

**Gene name**	**RAP-DB**	**MSU_RGAP7**	**Chromosome**	**Position**	**Size**	
					**CDS(bp)**	**Protein(aa)**
*OsHyPRP01*/*RCc3*	Os02g0662000	LOC Os02g44310	2	26,804,988–26,805,820	402	133
*OsHyPRP02*	Os02g0662100	LOC_Os02g44320	2	26,814,637–26,815,393	387	128
*OsHyPRP03*	Os03g0103100	LOC_Os03g01300	3	208,340–209,118	417	138
*OsHyPRP04*	Os03g0103200	LOC_Os03g01300	3	211,082–211,817	426	141
*OsHyPRP05*/*qLTG3-1*	Os03g0103300	LOC_Os03g01320	3	219,977–221,070	555	184
*OsHyPRP06*	Os04g0554500	LOC_Os04g46810	4	27,740,0640–27,740,809	393	130
*OsHyPRP07*	Os04g0554600	LOC_Os04g46820	4	27,743,339–27,743,963	396	131
*OsHyPRP08*	Os04g0554800	LOC_Os04g46830	10	27,753,369–27,754,530	414	137
*OsHyPRP09*	Os10g0349300	LOC_Os10g20830	10	10,563,123–10,563,841	414	137
*OsHyPRP10*	Os10g0349400	LOC_Os10g20840	10	10,566,488–10,566,901	414	137
*OsHyPRP11*	Os10g0349600	LOC_Os10g20860	10	10,570,876–10,573,989	402	133
*OsHyPRP12*	Os10g0349900	LOC_Os10g20890	10	10,587,298–10,587,959	381	126
*OsHyPRP13*/*RCc2*	Os10g0551800	LOC_Os10g40430	10	21,658,l92–21,658,818	420	146
*OsHyPRP14*	Os10g0551900	LOC_Os10g40440	10	21,662,454–21,663,219	429	142
*OsHyPRP15*	–	LOC_Os10g40460	10	21,667,261–21,667,939	396	131
*OsHyPRP16*	Os10g0552200	LOC_Os10g40470	10	21,67O,510–21,671,244	396	131
*OsHyPRP17*	Os10g0552300	LOC_Os10g40480	10	21,675,375–21,674,640	411	136
*OsHyPRP18*	Os10g0552600	LOC_Os10g40510	10	21,690,332–21,691,088	402	133
*OsHyPRP19*	Os10g0552700	LOC_Os10g40520	10	21,693,876–21,694,502	375	124
*OsHyPRP20*	Os10g0552800	LOC_Os10g40530	10	21,697,077–21,697,778	399	132
*OsHyPRP21*	Os10g055480	LOC_Os10g40614	10	21,786,108–21,787,038	504	167

*RCc3, RCc2, Xu et al. (1995)*.

*qLTG3-1; Fujino et al. (2008b)*.

The OsHyPRP genes showed high similarity to each other at amino acid alignments, 0.442–0.985 (Supplemental Table S2). The highest and lowest similarities occurred between *OsHyPRP06* and *OsHyPRP07* and between *OsHyPRP05* and *OsHyPRP19*, respectively. All, except for *OsHyPRP05* and *OsHyPRP21*, showed higher similarity, a mean of 0.730 ranging 0.641–0.985. These two OsHyPRP genes, *OsHyPRP05* and *OsHyPRP21*, had GRD and showed lower similarity with other members of the OsHyPRP genes, 0.498 and 0.539, respectively.

**Figure 2:**
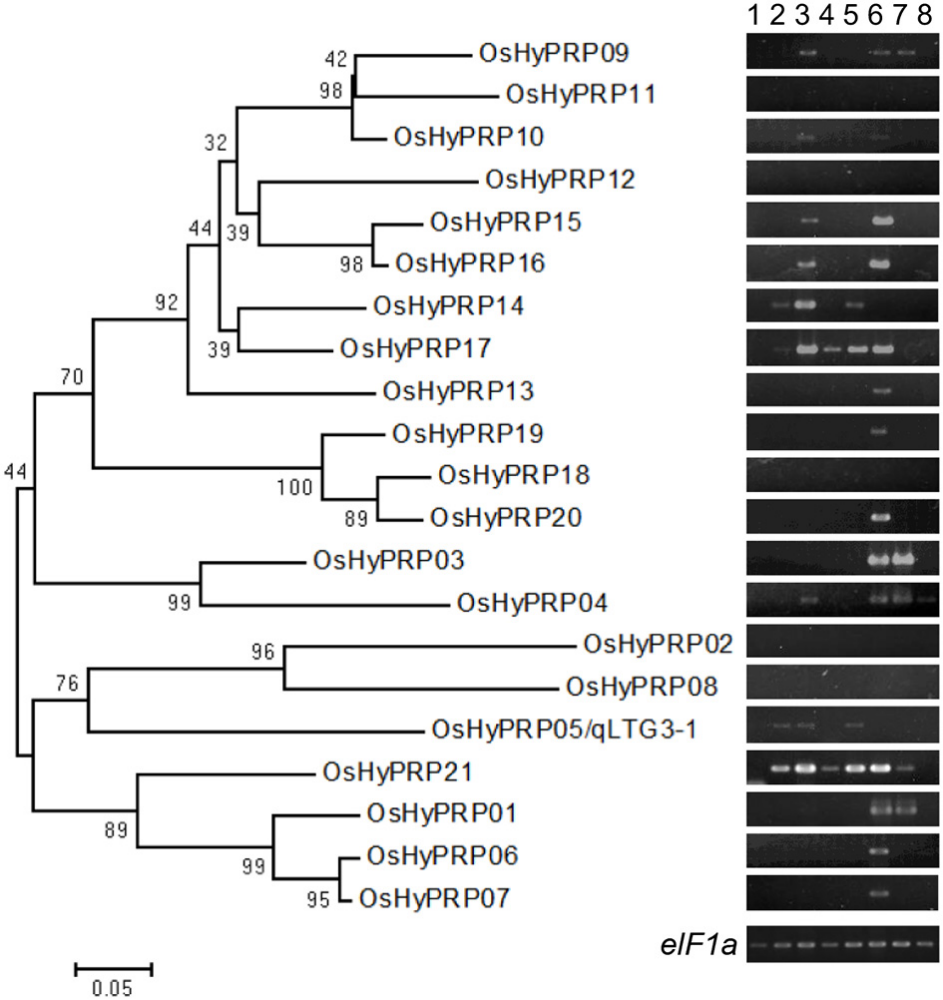
**Phylogenic relationships among the HyP/GRP gene family in rice with expression patterns**. A phylogenetic tree was constructed using neighbor-joining analysis in MEGA ver6. Bootstrap analysis values are shown at the nodal branches. The indicated scale represents 0.05 aa substitutions per site. RNA were extracted from various tissues; embryos during seed germination under 30°C conditions at 6 h after incubation (hai) (lane 1), 12 hai (lane 2), and 24 hai (lane 3), and under 15°C conditions at 1 day after incubation (dai) (lane 4) and 2 dai (lane 5), tip (lane 6) and basal (lane 7) of the root, and leaf (lane 8). *eIF1a* was used as a loading control.

The phylogenic tree of the OsHyPRP genes corresponded to tandem duplications and chromosome duplications (Figure 2). The differentiation of OsHyPRPs was mainly caused by amino acid substitutions within regions A and B. In contrast to the high similarity in region C, 0.920, those in regions A and B were 0.783 and 0.586, respectively. P/GRDs characterized each member of the OsHyP/GRP gene family.

### Expression profiles of the HyP/GRP gene family in rice

To determine the expression specificity of the HyP/GRP gene family in rice, semi-quantitative RT-PCR analysis was conducted using the RNA of Hokkaiwase extracted from various tissues: embryos during seed germination at different times, the tips and bases of the roots of 4-day-old seedlings, and the 3rd leaf blades of 3-week-old seedlings (Figure 2). Seventeen of the 21 members were expressed in the different tissues of Hokkaiwase. The expression of four genes, *OsHyPRP08, 11, 12*, and *18*, was negligible in these tissues. Gene expression levels varied among different tissues and times. *OsHyPRP01* showed a similar expression profile to *OsHyPRP03*, specifically to that in the root. *OsHyPRP06* showed a similar expression profile to *OsHyPRP07, 13, 19*, and *20*, specifically in the tip of the root. The gene expression of *OsHyPRP05* was detected after 12-h and 2-day incubations at 30 and 15°C during seed germination. The expression of eight genes, *OsHyPRP04, 09, 10, 14, 15, 16, 17*, and *21*, was detected during seed germination. These were located on chromosomes 3 and 10, suggesting that paralogous genes have similar gene expression patterns. The gene expression of *OsHyPRP21* was higher and earlier than that of *OsHyPRP05*. The overlapped and different gene expression patterns of the OsHyPRP genes suggested that OsHyPRP has redundant and different roles at the developmental stages in rice.

In contrast to the high similarity of amino acid alignments, sequence identity in 1 kb of the 5′ upstream regions from UTR was low, with a mean of 0.330 ranging 0.262–0.410 (Supplemental Table S3). Due to this low identity in the 5′ upstream regions, similar expression patterns may be controlled by a small number of *cis*-regulatory elements.

Similar expression patterns, but lower expression levels were detected in Kitaake and Hoshinoyume (Supplemental Figure S1). Since these varieties have different *qLTG3-1* alleles, they exhibited different growth stages from the start of the incubation. The results strongly suggested that the expression of these OsHyPRP genes is dependent on the developmental stage based on *qLTG3-1*.

### Sequence variations in *OsHyPRP21* in cultivated rice

Based on chromosomal locations and the glycine-rich motif, *OsHyPRP21* was considered to be a paralogous gene to *OsHyPRP05*. A total of 1128 nucleotides in the *OsHyPRP21* (*Os10g0554800*) gene were sequenced among 58 varieties in WRC. Compared with the sequence of Nipponbare as a reference allele (allele A), nine mutation events at nine sites, including deletions and substitutions, were detected (Supplemental Figure S2). Only three mutation events were detected in the coding region; two deletions at positions +95 and +193 and a single nonsynonymous substitution at position +139. These deletions occurred in-frame in the GRD, which contained Gly repeats with a Ser residue. As a result of the mutation events of these deletions, the repeat number varied. The A–G substitution at position +139 generated the amino acid substitution, Ser to Gly.

Based on these mutations, 4 different alleles were detected among WRC (Supplemental Figure S2, Table S4). Allele B, which was found in a single variety, was generated from intragenic recombinations between alleles A and C. Allele A included 30 varieties, while alleles C and D included 17 and 10 varieties, respectively. A clear relationship was observed between the allele types of *OsHyPRP21* and the cultivar group classification. Varieties of *japonica* and *aus* had allele A, while varieties of *indica* had alleles C and D.

Similar to *qLTG3-1* (*OsHyPRP05*) (Fujino and Sekiguchi, 2011), the almost completely conserved protein alignment of *OsHyPRP21* was identified. These results suggested that the function of *OsHyPRP21* is critical at least for seed germination, during which gene expression was detected.

### Diversity of the HyP/GRP gene family in monocots

The sequences of four genome-sequenced species, barley, maize, *Brachypodium*, and sorghum, were analyzed to determine the evolutional relationships among the HyP/GRP gene family in monocots. The results of a BLAST search with *qLTG3-1* as the query identified 12 genes in barley, 20 genes in maize, 10 genes in *Brachypodium*, and 28 genes in sorghum (Supplemental Figure S3, Table S4). All these species contained the HyP/GRP gene family, with predicted amino acid alignments ranging from 120 aa in barley to 325 aa in sorghum. Two HyP/GRP genes with a glycine rich motif was detected in barley, maize, and *Brachypodium*, while five were identified in sorghum. Species-specific tandem duplications occurred. In maize, four members of *ZmHyPRP05-08* and four members of *ZmHyPRP16-19* were tandem duplicated within the 52 kb region on chromosome 1 and the 197 kb region on chromosome 9, respectively. In *Brachypodium*, four members of *BdHyPRP02-05* were tandem duplicated within the 16 kb region on chromosomes 1. In sorghum, three members of *SbHyPRP02-04*, 11 members of *SbHyPRP08-18*, three members of *SbHyPRP20-22*, and four members of *SbHyPRP23-26* were tandem duplicated within the 22 kb region on chromosome 1, the 101 kb region on chromosome 4, the 19 kb region on chromosome 6, and the 52 kb region on chromosome 8, respectively.

**Figure 3:**
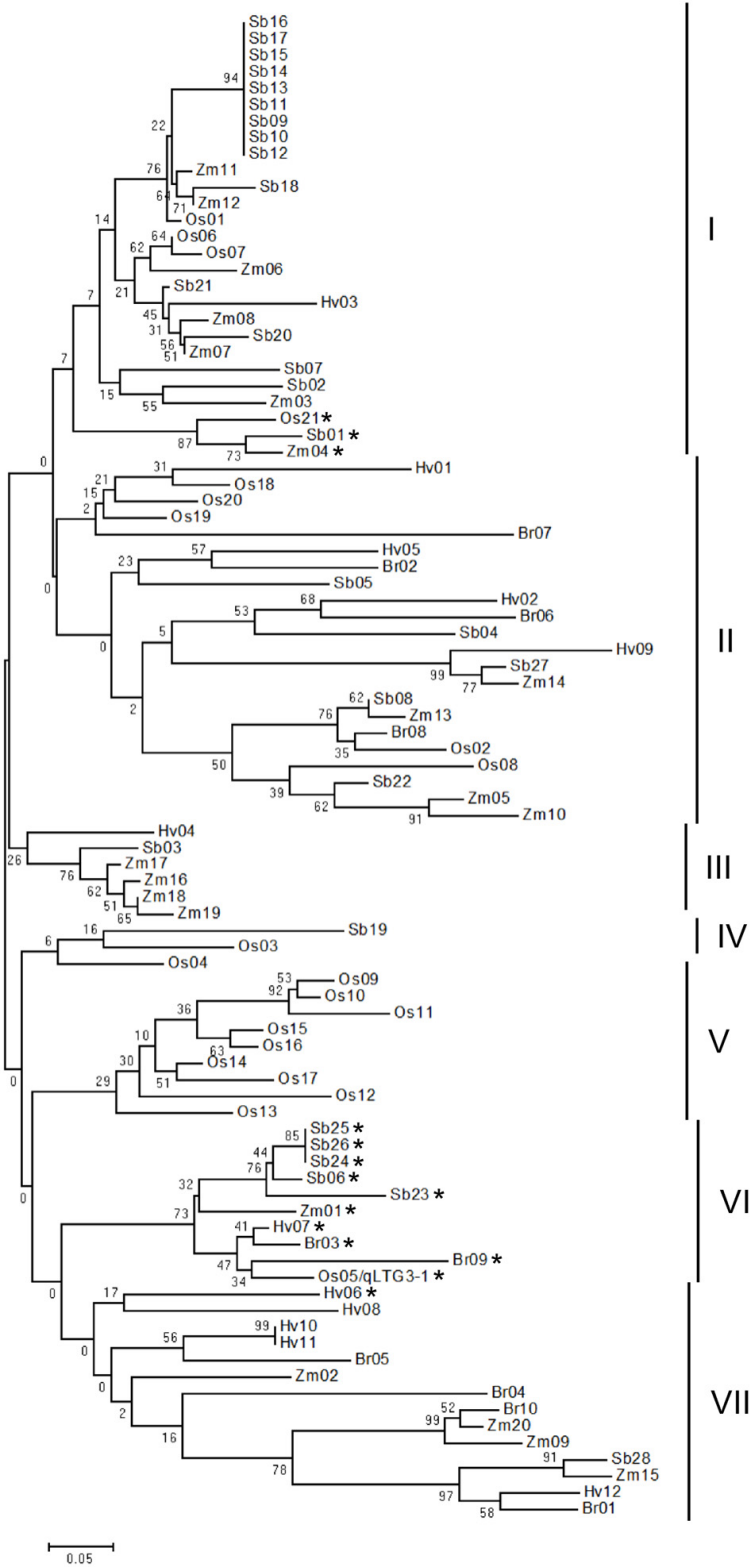
**Phylogenic tree of the HyP/GRP gene family in five monocots**. The phylogenetic tree was constructed using neighbor-joining analysis in MEGA ver6. Bootstrap analysis values are shown at the nodal branches. The indicated scale represents 0.05 aa substitutions per site. Vertical bars beside the tree indicate the seven-major-group classification. The asterisk indicates genes with a glycine-rich motif.

Phylogenetic analysis revealed that a total of 91 HyP/GRPs in these five monocots appeared to be divided into seven distinct groups (Figure 3). The main difference in the amino acid alignments in each group was the alignment of the proline/glycine-rich motif (Supplemental Figure S4). The nine members of OsHyPRP, which were tandem located on chromosome 10, formed a distinct group, group V, while HyP/GRPs from other groups were clustered together in each species.

In the phylogenic tree, *qLTG3-1* belonged to group VI with eight orthologous genes in monocots with marked similarity to *qLTG3-1*, with a mean of 0.738 and range of 0.568–1.000 (Supplemental Table S5). All members in group VI had GRD. Three genes with GRD, *OsHyPRP21, ZmHyPRP04*, and *SbHyPRP01*, formed a subcluster in group I. These results suggested that genes with a glycine-rich motif in group VI are the ancestor type, while those in group I are the paralogous type and diversified from the ancestor type. In the common ancestor of these monocots, *qLTG3-1* orthologous HyP/GRP genes with GRD may be generated and at least a single duplication may have occurred.

Ten conserved amino acids, LLALNLLFFT, were previously identified at the N-termini from a comparison of *qLTG3–1*-like proteins in plants (Fujino et al., 2008b). An allelic variation with a single amino acid substitution, LLALNLHFFT, was then identified, which had weak function on seed germination (Hori et al., 2010; Fujino and Iwata, 2011). The LLALNLL_F_ alignment was completely conserved in the nine genes for orthologous *qLTG3-1* (Supplemental Figure S5), suggesting that this alignment plays a significant role in the molecular function of *qLTG3-1*.

In contrast to the marked similarity of amino acid alignments among the *qLTG3-1* orthologous genes, sequence identity in 1 kb of the 5′ upstream regions from UTR among rice, *Brachypodium*, maize, and sorghum was low, with a mean of 0.422 and range of 0.274–0.756 (Supplemental Table S6). Among the three conserved motifs expected, two conserved motifs were identified as a cis-regulatory motif, AGCT repeat, and ATGC repeat (Supplemental Figures S6, S7). The ATGC repeat had a CATGCA sequence, called the RY element, which was previously shown to be crucial for transactivation through ABI3/VP1-like B3-domain proteins (Ezcurra et al., 2000; Reidt et al., 2000) and has been predominantly detected in seed-specific promoters (Lelievre et al., 1992). Therefore, the RY element was found in *Brachypodium*, maize, and sorghum, but not in rice.

Phylogenetic analysis of the HyP/GRP genes in monocots strongly suggested that the expansion of the HyP/GRP gene family occurred from the latest common ancestor of the monocots of these five species. These results indicated that orthologous HyP/GRP genes have the same function in each species. Whole genome duplications and tandem duplications after the differentiation of these species may contribute to the expansion of HyP/GRP genes as a gene family.

### Collinearity of chromosomal regions around *qLTG3-1* orthologous genes

To clarify whether the genes in group VI originated from a common ancestral gene, we compared the chromosomal locations of these genes. *OsHyPRP05*/*qLTG3-1* was located on rice chromosome 3. Others were *BrHyPRP03* on *Brachypodium* chromosome 1, *HvHyPRP07* on barley chromosome 4, *ZmHyPRP01* on maize chromosome 1, and *SbHyPRP06* on sorghum chromosome 1. A previous study reported that these chromosomes had homologous relationships (Abrouk et al., 2010). These genes among monocots were orthologous to rice *qLTG3-1*, indicating that these genes within group VI were evolutionary related through a common ancestral gene on chromosome A7 from a putative ancestor in the proposed angiosperm evolutionary models (Abrouk et al., 2010).

**Figure 4:**
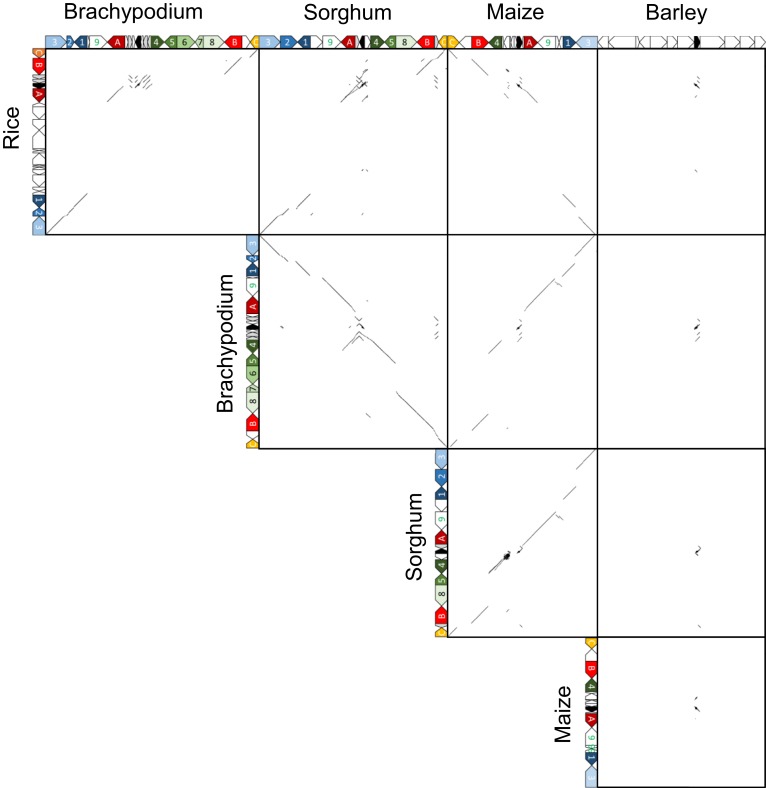
**Dot plot analysis to identify CDS regions around *qLTG3-1* orthologous genes among monocots**.

**Figure 5:**
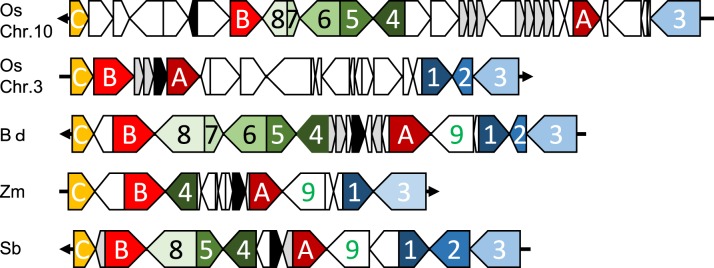
**Collinearity around *qLTG3-1* orthologous genes in monocots**. Os, rice; Bd, Brachypodium; Zm, maize; Sb, sorghum; Black box, *qLTG3-1* orthologous gene; Gray box, HyPRP gene; White box, unique genes for each species; A, GRAS transcription factor; B, Similar to Nitrate transporter; C, Expansin precursor; 1, Tubulin; 2, unknown protein; 3, unknown protein; 4, Exostosin-like; 5, Flavin-containing monooxygenase; 6, Unknown protein; 7, unknown protein; 8, oxysterol-binding protein; 9, Signal recognition particle receptor.

Orthologous chromosomal regions were aligned and compared to identify the conservation of micro-synteny around *qLTG3-1* and its orthologous genes (Supplemental Figure S8). However, only a small region was conserved among the monocots. The coding regions (CDSs) were then aligned and compared. Conserved gene alignments were detected among these species, except for barley, and high homology was detected between homologous CDSs (Figures 4, 5). Although the number of tandem duplicated HyP/GRP genes differed, the gene order was highly conserved among maize, sorghum, and *Brachypodium*.

**Figure 6:**
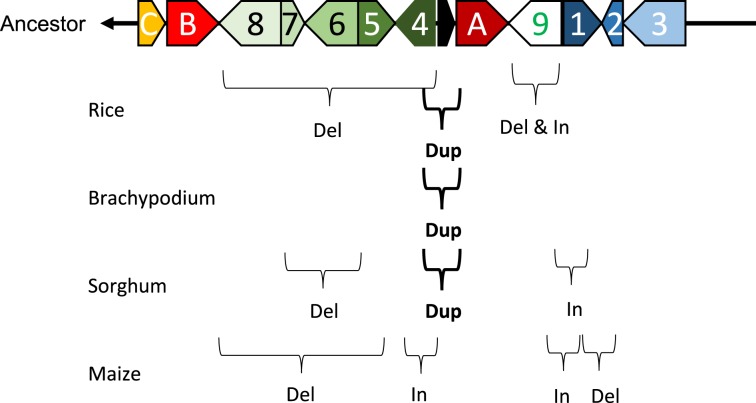
**Model of structural changes in chromosomal regions around *qLTG3-1* orthologous genes during monocot diversification**. Del, Dup, and In indicate deletion, duplication, and insertion, respectively. The ancestor type is modeled based on collinearity among monocots.

Marked genomic collinearity was observed among *Brachypodium*, sorghum, and maize, while collinearity could not be detected in barley. The gene order in the ancestor genome was proposed based on collinearity (Figure 6). According to the gene order, rearrangements including duplications, deletions, and insertions were involved in each monocot genome, which occurred in similar chromosomal regions.

## Conclusions

HyP/GRP functions have been closely related to plant development and responses to biotic and abiotic stresses. These are involved in important agronomical traits. A clearer understanding of the molecular functions of these genes may contribute to the production of stable plants, such as low temperature germinability in rice (Fujino et al., 2004, 2008b). A comprehensive overview of the HyP/GRP gene family in rice and other monocots has been presented in this study. Based on a comparison of phylogenetic relationships, chromosomal localizations, similarity/identity, and collinearity of genomic structures around *qLTG3-1* orthologous genes among monocots, the *qLTG3-1* orthologous genes may have conserved gene function among rice, *Brachypodium*, maize, and sorghum.

*qLTG3-1* was first identified as the genetic locus controlling low-temperature germinability in rice (Fujino et al., 2004). It was then cloned and its role was characterized in seed germination by histological observations and genome-wide expression analyses (Fujino et al., 2008b). Although the molecular function of the gene remains unclear, it may be involved in weakening the tissue covering the embryo. In barley, the coleorhiza covering the root plays a major role in causing dormancy by acting as a barrier to root emergence (Barrero et al., 2009). Comparative transcriptomics revealed that orthologous genes in syntenic genomic blocks are more likely to share correlated expression patterns (Davidson et al., 2012). The anatomical features of monocots seeds, especially the embryo and its surrounding tissue, were different. Therefore, seed germination may be controlled under the same genetic pathway including *qLTG3-1* and its orthologous genes.

Functional genomics in one species can be hypothesized to occur in syntenic regions in another species, namely, translational functional genomics (Paterson et al., 2010). Although rice separated from maize and sorghum ~50 million years ago (Mya) and from wheat and barley ~40 Mya, their common evolutionary history can be traced by the collinear order of genetic markers across their chromosome (Rice Chromosome 3 Sequencing Consortium, 2005). In the present study, in addition to collinearity around the *qLTG3-1* orthologous genes, a phylogenetic tree revealed that HyP/GRPs from different species were clustered together. A more detailed characterization of members of the OsHyPRP gene family may facilitate not only a deeper understanding of the molecular functions of HyP/GRPs, but also improved traits.

## Author contributions

Conceived, designed the experiments, and wrote the manuscript: Kenji Fujino. Performed the experiments: Mari Obara, Koji Sato, Kenji Fujino. Analyzed the data: Mari Obara, Koji Sato, Kenji Fujino.

### Conflict of interest statement

The authors declare that the research was conducted in the absence of any commercial or financial relationships that could be construed as a potential conflict of interest.

## References

[B1] Abrouk M. Murat F. Pont C. Messing J. Jackson S. Faraut T. (2010). Palaeogenomics of plants: synteny-based modelling of extinct ancestors. Trends Plant Sci. 15, 479–487 10.1016/j.tplants.2010.06.001 20638891

[B2] Adams K. L. Wendel J. F. (2005). Polyploidy and genome evolution in plants. Curr. Opin. Plant Biol. 8, 135–141 10.1016/j.pbi.2005.01.001 15752992

[B3] Ahn S. Tanksley S. D. (1993). Comparative linkage maps of the rice and maize genomes. Proc. Natl. Acad. Sci. U.S.A. 90, 7980–7984 10.1073/pnas.90.17.7980 8103599 PMC47271

[B4] Barrero J. M. Talbot M. J. White R. G. Jacobsen J. V. Gubler F. (2009). Anatomical and transcriptomic studies of the coleorhiza reveal the importance of this tissue in regulating dormancy in barley. Plant Physiol. 150, 1006–1021 10.1104/pp.109.137901 19386806 PMC2689963

[B5] Blanco-Portales R. López-Raéz J. A. Bellido M. L. Moyano E. Dorado G. González-Reyes J. A. (2004). A strawberry fruit-specific and ripening-related gene codes for a HyPRP protein involved in polyphenol anchoring. Plant Mol Biol. 55, 763–780 10.1007/s11103-005-1966-z 15604715

[B6] Bouton S. Viau L. Lelièvre E. Limami A. M. (2005). A gene encoding a protein with a proline-rich domain (MtPPRD1), revealed by suppressive subtractive hybridization (SSH), is specifically expressed in the *Medicago truncatula* embryo axis during germination. J. Exp. Bot. 56, 825–832 10.1093/jxb/eri077 15689340

[B7] Davidson R. M. Gowda M. Moghe G. Lin H. Vaillancourt B. Shiu S. H. (2012). Comparative transcriptomics of three Poaceae species reveals patterns of gene expression evolution. Plant J. 71, 492–502 10.1111/j.1365-313X.2012.05005.x 22443345

[B8] Deutch C. E. Winicov I. (1995). Post-transcriptional regulation of a salt-inducible alfalfa gene encoding a putative chimeric proline-rich cell wall protein. Plant Mol. Biol. 27, 411–418 10.1007/BF00020194 7888629

[B9] Dingkuhn M. De Datta S. K. Pamplona R. Javellana C. Schnier H. F. (1992). Effect of late-season N-fertilization on photosynthesis and the yield of transplanted and direct-seeded tropical flooded rice. 2. A canopy stratification study. Field Crop. Res. 28, 235–249 10.1016/0378-4290(92)90043-9

[B10] Dvoráková L. Cvrcková F. Fischer L. (2007). Analysis of the hybrid proline-rich protein families from seven plant species suggests rapid diversification of their sequences and expression patterns. BMC Genomics 8:412 10.1186/1471-2164-8-412 17997832 PMC2216038

[B11] Ezcurra I. Wycliffe P. Nehlin L. Ellerström M. Rask L. (2000). Transactivation of the *Brassica napus* napin promoter by ABI3 requires interaction of the conserved B2 and B3 domains of ABI3 with different cis-elements: B2 mediates activation through an ABRE, whereas B3 interacts with an RY/G-box. Plant J. 24, 57–66 10.1046/j.1365-313x.2000.00857.x 11029704

[B12] Fujino K. Iwata N. (2011). Selection for low-temperature germinability on the short arm of chromosome 3 in rice cultivars adapted to Hokkaido, Japan. Theor. Appl. Genet. 123, 1089–1097 10.1007/s00122-011-1650-4 21744228

[B13] Fujino K. Matsuda Y. (2010). Genome-wide analysis of genes targeted by *qLTG3-1* controlling low-temperature germinability in rice. Plant Mol. Biol. 72, 137–152 10.1007/s11103-009-9559-x 19851874

[B14] Fujino K. Matsuda Y. Ozawa K. Nishimura T. Koshiba T. Fraaije M. W. (2008a). *NARROW LEAF 7* controls leaf shape mediated by auxin in rice. Mol. Genet. Genomics 279, 499–507 10.1007/s00438-008-0328-3 18293011

[B15] Fujino K. Sekiguchi H. (2011). Origins of functional nucleotide polymorphisms in a major quantitative locus, *qLTG3-1*, controlling low-temperature germinability in rice. Plant Mol. Biol. 75, 1–10 10.1007/s11103-010-9697-1 20960223

[B16] Fujino K. Sekiguchi H. Kiguchi T. (2005). Identification of an active transposon in intact rice plants. Mol. Genet. Genomics 273, 150–157 10.1007/s00438-005-1131-z 15803319

[B17] Fujino K. Sekiguchi H. Matsuda Y. Sugimoto K. Ono K. Yano M. (2008b). Molecular identification of a major quantitative trait locus, *qLTG3-1*, controlling low-temperature germinability in rice. Proc. Natl. Acad. Sci. U.S.A. 105, 12623–12628 10.1073/pnas.0805303105 18719107 PMC2527961

[B18] Fujino K. Sekiguchi H. Sato T. Kiuchi H. Nonoue Y. Takeuchi Y. (2004). Mapping of quantitative trait loci controlling low-temperature germinability in rice (*Oryza sativa* L.). Theor. Appl. Genet. 108, 794–799 10.1007/s00122-003-1509-4 14624339

[B18a] Fujino K. Wu J. Sekiguchi H. Ito T. Izawa T. Matsumoto T. (2010). Multiple introgression events surrounding the *Hd1* flowering-time gene in cultivated rice, *Oryza sativa* L. Mol. Genet. Genomics 284, 137–146 10.1007/s00438-010-0555-2 20607290

[B19] Goodwin W. Pallas J. A. Jenkins G. I. (1996). Transcripts of a gene encoding a putative cell wall-plasma membrane linker protein are specifically cold-induced in *Brassica napus*. Plant Mol. Biol. 31, 771–781 10.1007/BF00019465 8806408

[B20] Gu Z. Steinmetz L. M. Gu X. Scharfe C. Davis R. W. Li W. H. (2003). Role of duplicate genes in genetic robustness against null mutations. Nature 421, 63–66 10.1038/nature01198 12511954

[B21] Hamilton J. P. Buell C. R. (2012). Advances in plant genome sequencing. Plant J. 70, 177–190 10.1111/j.1365-313X.2012.04894.x 22449051

[B22] He C. Y. Zhang J. S. Chen S. Y. (2002). A soybean gene encoding a proline-rich protein is regulated by salicylic acid, an endogenous circadian rhythm and by various stresses. Theor. Appl. Genet. 104, 1125–1131 10.1007/s00122-001-0853-5 12582622

[B23] Holk A. Klumpp L. Scherer G. F. (2002). A cell wall protein down-regulated by auxin suppresses cell expansion in *Daucus carota* (L.). Plant Mol. Biol. 50, 295–305 10.1023/A:1016052613196 12175021

[B24] Hori K. Sugimoto K. Nonoue Y. Ono N. Matsubara K. Yamanouchi U. (2010). Detection of quantitative trait loci controlling pre-harvest sprouting resistance by using backcrossed populations of *japonica* rice cultivars. Theor. Appl. Genet. 120, 1547–1557 10.1007/s00122-010-1275-z 20145904 PMC2859223

[B25] Hughes A. L. (1994). The evolution of functionally novel proteins after gene duplication. Proc. Biol. Sci. 256, 119–124 10.1098/rspb.1994.0058 8029240

[B26] International Rice Genome Sequencing Project. (2005). The map-based sequence of the rice genome. Nature 436, 793–800 10.1038/nature03895 16100779

[B27] Josè-Estanyol M. Ruiz-Avila L. Puigdomènech P. (1992). A maize embryo-specific gene encodes a proline-rich and hydrophobic protein. Plant Cell 4, 413–423 10.1105/tpc.4.4.413 1498600 PMC160141

[B28] Jung H. W. Tschaplinski T. J. Wang L. Glazebrook J. Greenberg J. T. (2009). Priming in systemic plant immunity. Science 324, 89–91 10.1126/science.1170025 19342588

[B29] Kojima Y. Ebana K. Fukuoka S. Nagamine T. Kawase M. (2005). Development of an RFLP-based rice diversity research set of germplasm. Breed. Sci. 55, 431–440 10.1270/jsbbs.55.431

[B30] Lelievre J. M. Oliveira L. O. Nielsen N. C. (1992). 5'CATGCAT-3′ elements modulate the expression of glycinin genes. Plant Physiol. 98, 387–391 10.1104/pp.98.1.387 16668640 PMC1080194

[B31] Lynch M. Force A. (2000). The probability of duplicate gene preservation by subfunctionalization. Genetics 154, 459–473 10629003 10.1093/genetics/154.1.459 PMC1460895

[B32] Murray M. G. Thompson W. F. (1980). Rapid isolation of high molecular weight plant DNA. Nucleic Acids Res. 8, 4321–4325 10.1093/nar/8.19.4321 7433111 PMC324241

[B33] Paterson A. H. Bowers J. E. Bruggmann R. Dubchak I. Grimwood J. Gundlach H. (2009). The *Sorghum bicolor* genome and the diversification of grasses. Nature 457, 551–556 10.1038/nature07723 19189423

[B34] Paterson A. H. Freeling M. Haibao Tang H. Wang X. (2010). Insights from the comparison of plant genome sequences. Ann. Rev. Plant Biol. 61, 349–372 10.1146/annurev-arplant-042809-112235 20441528

[B35] Peterson M. L. Jones D. B. Rutger J. N. (1978). Cool Temperature Screening of Rice Lines for Seedling Vigor. Il Riso, Vol. 27 Milan: Ente Nazionale Risi

[B36] Priyanka B. Sekhar K. Reddy V. D. Rao K. V. (2010). Expression of pigeonpea hybrid-proline-rich protein encoding gene (CcHyPRP) in yeast and *Arabidopsis* affords multiple abiotic stress tolerance. Plant Biotechnol. J. 8, 76–87 10.1111/j.1467-7652.2009.00467.x 20055960

[B37] Reidt W. Wohlfarth T. Ellerström M. Czihal A. Tewes A. Ezcurra I. (2000). Gene regulation during late embryogenesis: the RY motif of maturation-specific gene promoters is a direct target of the FUS3 gene product. Plant J. 21, 401–408 10.1046/j.1365-313x.2000.00686.x 10758492

[B38] Rice Chromosome 3 Sequencing Consortium. (2005). Sequence, annotation, and analysis of synteny between rice chromosome 3 and diverged grass species. Genome Res. 15, 1284–1291 10.1101/gr.3869505 16109971 PMC1199543

[B39] Scannell D. R. Byrne K. P. Gordon J. L. Wong S. Wolfe K. H. (2006). Multiple rounds of speciation associated with reciprocal gene loss in polyploid yeasts. Nature 440, 341–345 10.1038/nature04562 16541074

[B40] Schnable P. S. Ware D. Fulton R. S. Stein J. C. Wei F. Pasternak S. (2009). The B73 maize genome: complexity, diversity, and dynamics. Science 326, 1112–1115 10.1126/science.1178534 19965430

[B41] Sjödin P. Hedman H. Kruskopf Osterberg M. Gustafsson S. Lagercrantz U. Lascoux M. (2008). Polymorphism and divergence at three duplicate genes in *Brassica nigra*. J. Mol. Evol. 66, 581–590 10.1007/s00239-008-9108-2 18470551

[B42] Tan J. Zhuo C. Guo Z. (2013). Nitric oxide mediates cold- and dehydration-induced expression of a novel MfHyPRP that confers tolerance to abiotic stress. Physiol. Plant. 149, 310–320 10.1111/ppl.12032 23387330

[B43] The International Barley Genome Sequencing Consortium. (2012). A physical, genetic and functional sequence assembly of the barley genome. Nature 491, 711–716 10.1038/nature11543 23075845

[B44] The International Brachypodium Initiative. (2010). Genome sequencing and analysis of the model grass *Brachypodium distachyon*. Nature 463, 763–768 10.1038/nature08747 20148030

[B45] Throude M. Bolot S. Bosio M. Pont C. Sarda X. Quraishi U. M. (2009). Structure and expression analysis of rice paleo duplications. Nucleic Acids Res. 37, 1248–1259 10.1093/nar/gkn1048 19136467 PMC2651813

[B46] Wang X. Shi X. Hao B. Ge S. Luo J. (2005). Duplication and DNA segmental loss in the rice genome: implications for diploidization. New Phytol. 165, 937–946 10.1111/j.1469-8137.2004.01293.x 15720704

[B47] Weyman P. D. Pan Z. Feng Q. Gilchrist D. G. Bostock R. M. (2006). *DEA1*, a circadian- and cold-regulated tomato gene, protects yeast cells from freezing death. Plant Mol. Biol. 62, 547–559 10.1007/s11103-006-9039-5 16897467

[B48] Wu H. M. Zou J. May B. Gu Q. Cheung A. Y. (1993). A tobacco gene family for flower cell wall proteins with a proline-rich domain and a cysteine-rich domain. Proc. Natl. Acad. Sci. U.S.A. 90, 6829–6833 10.1073/pnas.90.14.6829 8341705 PMC47026

[B49] Xu Y. Buchholz W. G. DeRose R. T. Hall T. C. (1995). Characterization of a rice gene family encoding root-specific proteins. Plant Mol. Biol. 27, 237–248 10.1007/BF00020180 7888615

[B50] Yeom S. I. Seo E. Oh S. K. Kim K. W. Choi D. (2012). A common plant cell-wall protein HyPRP1 has dual roles as a positive regulator of cell death and a negative regulator of basal defense against pathogens. Plant J. 69, 755–768 10.1111/j.1365-313X.2011.04828.x 22023393

[B51] Zhang Y. Schläppi M. (2007). Cold responsive EARLI1 type HyPRPs improve freezing survival of yeast cells and form higher order complexes in plants. Planta 227, 233–243 10.1007/s00425-007-0611-2 17786468

