# Supplementary material for: Diversification of the plant-specific hybrid glycine-rich protein (HyGRP) genes in cereals
Source: Front Plant Sci. 2014 Sep 24;5:489. doi: 10.3389/fpls.2014.00489 (PMC4174136; doi:10.3389/fpls.2014.00489)
Supplement: Supplementary file 1 [file DataSheet1.PDF]

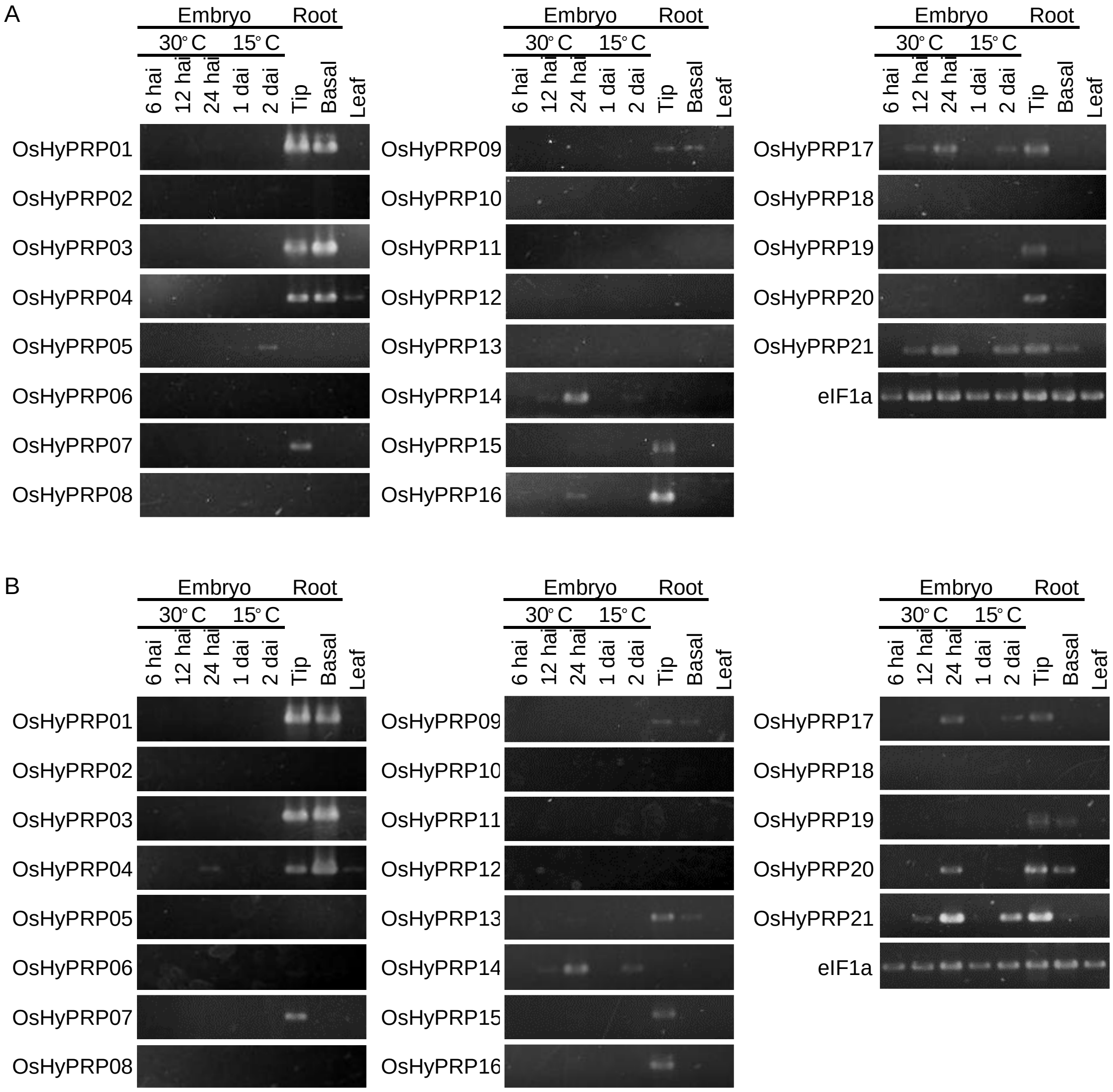

Supplemental FIGURE S1 Expression of OsHyPRP genes by semi-quantitative RT-PCR analysis. For RNA extracted from embryos during seed germination under 15 and 30 °C conditions, the root, and leaf were used. *eIF1a* was used as a loading control. (A) Kitaake. (B) Hoshinoyume.

A

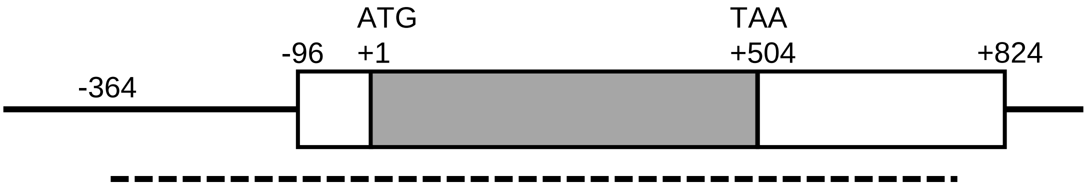

B

Allele group	Position -161	-159	-114	-54	-25	95	139	193	657
A	-	C	C_5	T	G	$(GCG)_5$	A	-	G
B	-	T	C_5	T	T	$(GCG)_4$	G	12	G
C	-	T	C_5	T	T	$(GCG)_4$	G	12	T
D	3	C	C_4	G	T	$(GCG)_4$	A	12	T
Category	D	N	D	N	N	D	R	D	N
						G	S47G	GSSG	

C

```
Allele A    MASSRASASCALFLALNLLLFAITTACPSCGSGGGGGHGHYGGGGSSGGGGGYGGGSGGY 60
Allele B    MASSRASASCALFLALNLLLFAITTACPSCGSGGGG-HGHYGGGGSGGGGGGYGGGSGGY 59
Allele C    MASSRASASCALFLALNLLLFAITTACPSCGSGGGG-HGHYGGGGSGGGGGGYGGGSGGY 59
Allele D    MASSRASASCALFLALNLLLFAITTACPSCGSGGGG-HGHYGGGGSSGGGGGYGGGSGGY 59
            ************************************ *********.*************

Allele A    GGGGSSGGGYGGGGGGSSTSGWYGKCPTDALKLGVCANVLDLIKAKAGVPATEPCCPLLNG 120
Allele B    GGG----GGYGGGGGGSSTSGWYGKCPTDALKLGVCANVLDLIKAKAGVPATEPCCPLLNG 115
Allele C    GGG----GGYGGGGGGSSTSGWYGKCPTDALKLGVCANVLDLIKAKAGVPATEPCCPLLNG 115
Allele D    GGG----GGYGGGGGGSSTSGWYGKCPTDALKLGVCANVLDLIKAKAGVPATEPCCPLLNG 115
            ***    *****************************************************

Allele A    LVDLEAAVCLCTAIKANVLGINLNLPIHLSLILNFCGKGVPTGFMCS 167
Allele B    LVDLEAAVCLCTAIKANVLGINLNLPIHLSLILNFCGKGVPTGFMCS 162
Allele C    LVDLEAAVCLCTAIKANVLGINLNLPIHLSLILNFCGKGVPTGFMCS 162
Allele D    LVDLEAAVCLCTAIKANVLGINLNLPIHLSLILNFCGKGVPTGFMCS 162
            ***********************************************
```

Supplemental FIGURE S2 Nucleotide changes in the 1,128-bp OsHyPRP21 region among rice cultivars. (A) The structure of OsHyPRP21. White and gray boxes indicate UTR and coding regions, respectively. The dotted line indicates sequenced regions. **(B)** Allele classification based on polymorphisms. Categories D, N, and R indicate deletion, non-coding site, and replacement, respectively. The number indicates the size (bp) of deletions. **(C)** Sequence alignment of four OsHyPRP21 alleles.

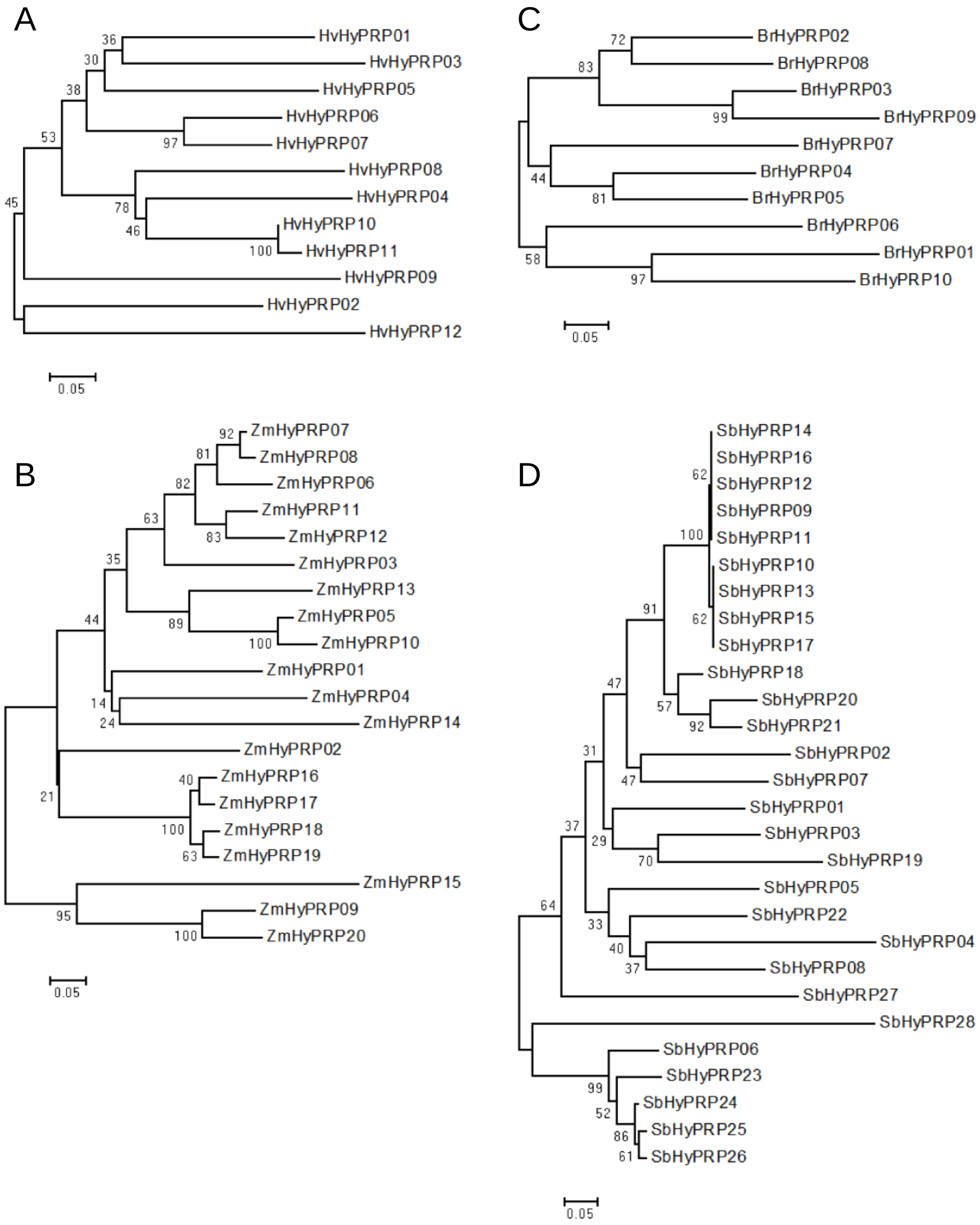

Supplemental FIGURE S3 Phylogenic tree of the HyP/GRP gene family in barley (A), maize (B), *Brachypodium* (C), and sorghum (D). The phylogenetic tree was constructed using neighbor-joining analysis in MEGA ver6. Bootstrap analysis values are shown at the nodal branches. The indicated scale represents 0.05 aa substitutions per site.

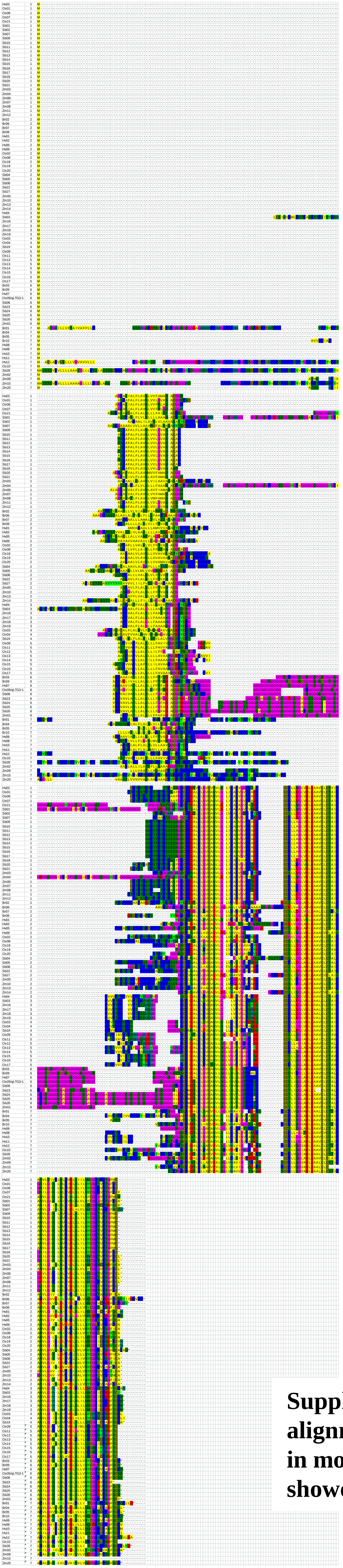

Supplemental FIGURE S4 Sequence alignment of the HyP/GRP gene family in monocots. Different amino acids are showed by different colors

```
HvHyPRP07   MAKKAAVIATLLALNLLFFTFADACGCHCGSCPSPGGGG------------GGGGGGGGGGGGGGSGGGG
BrHyPRP03   MAKKAAAIATLLALNLLFFTFSDACGCHCG------------------------------GGGSGPGGGS
BrHyPRP09   MAKKVELIVALLALNLLFFTFSDASRRSYPPAAGGGGGG----------------GGGNGGGGGNCGGGN
ZmHyPRP01   MAKK---VATLLALNLLFFAFADACGCRCGGSCPSPGGG--------------GGGGSGGGGGGSGGGGS
SbHyPRP06   MAKKVALVASLLALNLLLFGFADACGCRCGGSCPSPPGG--------------GGGGGGGGGGG------
SbHyPRP23   MAKKVAVLATLLALNLLLFSFGEACGYDGAGSGGTGGGS--------------GGGGSGAGGSGGAGSGG
SbHyPRP24   MVKKVAVLATLLALNLLLFGFADACGYDGGGSGGTGGSG---TSGGGSGSGGTSGGGSGGGGSGGGGSGG
SbHyPRP25   MVKKVAVLATLLALNLLLFGFADACGYDGGGSGGTGGSG---TSGGGSGSGGTSGGGSGGGGSGGGGSGG
SbHyPRP26   MVKKVAVLATLLALNLLLFGFADACGYDGGGSGGTGGSGGGGTGGGGSGSGGTGGGGSVGGGSGGGGSGG
OsHyPRP05   MATKAGVIATLLALNLHFFTFSDACGCQC GSCPSPGGG--------------GGGGGGGGGGG------

HvHyPRP07   GGSGGSGGSGGGGSGGGGSGGGGSGGGGSGGGGSGGGGSGGGG---------------------------
BrHyPRP03   GGSGGSGGSGGGGSGGGGSGSGGGGSGGGGGGGSG-----------------------------------
BrHyPRP09   CGGGGSGNCGGTGNGGGGTCGGGNCGNCGGGGNGGGGNNGGGG---------------------------
ZmHyPRP01   GGGGSGGGSGGGGSGGGGSGGGGSSGGGGGSGGGGGGGSGGGG--------------------------G
SbHyPRP06   ------------GSGGGGSGGGGGSGGGGSGGGGSGGGGGGGG--------------------------G
SbHyPRP23   DGSGGVGGSSGGGSSGGGSGRGGISGGGSGAGGSSGGSSGAGG------------------------SGS
SbHyPRP24   GGNGGAGGSSGGGSIGGGSGGRGSGGGGSGAGGSGGGGSGAGGNGGGAGGSGGGGSGSGGSGSGGGGSGS
SbHyPRP25   GGNGGAGGSSGGGSIGGGSGGRGSGGGGSGAGGSGGGGSGAGGNGGGAGGSGGGGSGSGGSGSGGGGSGS
SbHyPRP26   GGSGGARGSSGGGSIGGGSGGRGSGGGGSGAGGSGGGGSGAGGNGGGAGGSGGGGSGSGGSGSGGGGSGS
OsHyPRP05   --GGRGGGGGSGGGSGGGGGSGGGGSGGGGSGGGGSGGGGGGG--------------------------S

HvHyPRP07   -GSGGLARCPIDALKLGVCANVLNGLINLQLGTPPKKPCCTLIQGLADLEAAVCLCTALKANILGI-NLN
BrHyPRP03   --SGGLGRCPIDALKLGVCANVLNGLINLELGTPPKKPCCTLIQGLADLEAAVCLCTALRANILGI-NLN
BrHyPRP09   -NGGNSSSCPIDALKLGVCANLLNGLLNLQLGTPPALPCCSVIRGLLDLEAAVCLCTALRANILGIINLN
ZmHyPRP01   GGGGTSGRCPVDALKLGVCANLLNGLINATLGTPPRTPCCTLIQGLADLEAAVCLCTVLRANVLGI-NLN
SbHyPRP06   GGGGTRGRCPVDTLKLGVCANVLNGLINVNLGTPPRTPCCTLIQGLADLEAAVCLCTALRANILGI-NLN
SbHyPRP23   GGGGTLGRCPMDTLKLGVCANVLNGLINLNLGTPPKTPCCTLIQGLADLEL--CLCTALKANILGI-NLN
SbHyPRP24   GGGGALERCPMDTLKLGVCANVLNGLINVTLGTPPKTPCCTLIQGLADLEAAVCLCTALKANILGI-NLN
SbHyPRP25   GGGGALERCPMDTLKLGVCANVLNGLINVTLGTPPKMPCCTLIQGLADLEAAVCLCTALRANILGI-NLN
SbHyPRP26   GGGGALERCPMDTLKLGVCANVLNGLINVTLGTPPKTPCCTLIQGLADLEAAVCLCTALRANILGI-NLN
OsHyPRP05   GGGGGGGRCPIDTLKLGVCANVLNGLINVQLGTPPRQPCCSLIQGLADLEAAVCLCTALRANILGI-NLN

HvHyPRP07   VPIDLSLLVNYCGKNVPAGFQCPS
BrHyPRP03   VPIDLSLLVNYCGKRVPTGFQCP-
BrHyPRP09   IPIDLSLLINYCGGTVPSGFQCN-
ZmHyPRP01   LPINLSLLVNYCGRRVPSGFQCF-
SbHyPRP06   LPINLSLLVNYCGRRVPSGFQCSS
SbHyPRP23   LPINLSLIANYCGKDVPSGF----
SbHyPRP24   LPINLSLLVNYCGKDVPSGFQCS-
SbHyPRP25   LPINLSLLVNYCGKDVPSGFQCS-
SbHyPRP26   LPINLSLLVNYCGKDVPSGFQCS-
OsHyPRP05   LPINLSLLVNYCGRSVPSGFQCSN
```

Supplemental FIGURE S5 Sequence alignment of qLTG3-1 orthologous genes in monocots.

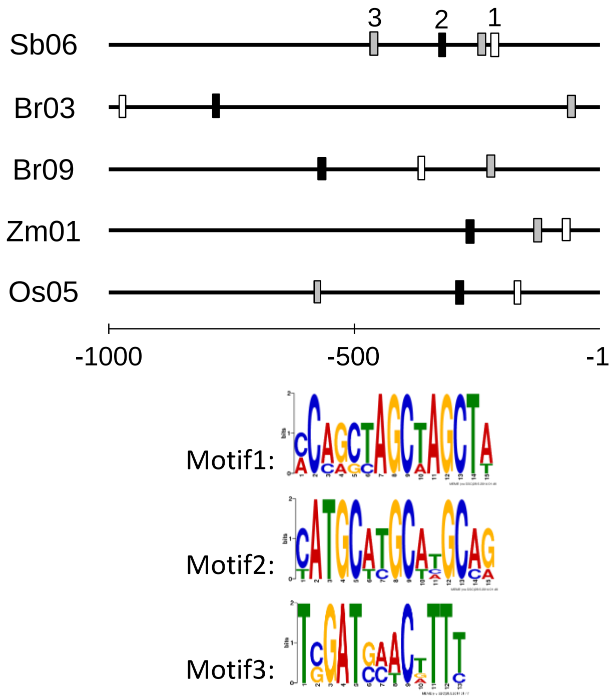

Supplemental FIGURE S6 Conserved motifs among *qLTG3-1* orthologous genes in monocots. The motif analysis was performed by MEME (http://meme.nbcr.net/meme/) according to the default setting. White, black, and gray boxes indicate motifs 1, 2, and 3, respectively. The number shows the distance from the start of 5' UTR.

Motif 1

```
OsHyPRP05 F -192 GTACTCCAGG  CCAGCTAGCTAGCTA  GGTCACTAGT -158
BrHyPRP09 R -349 ACAACGTGCC  CCAGCTAGCTAGCTA  GCTAGCTTGC -383
SbHyPRP06 F -226 CTTCCTCGTG  CCAGCTAGCTAGCTT  TTCACCTTTC -192
BrHyPRP03 F -990 CCAAAACCTC  ACCGCTAGCTAGCTA  AGCTGTGTGT -956
ZmHyPRP01 F -87  AGCAGTTACT  ACAAGCAGCAAGCTA  CTCCTAACTA -53
```

Motif 2

```
OsHyPRP05   F -306 TATATATATA  TATGCTCGCTAGCAG  AGTACTTGGC -272
BrHyPRP09   R -557 TCTCCCATGA  CATGCATGCATGCCA  ATAAGTTTTT -591
SbHyPRP06   F -335 AATTGCAAAG  CATGCATGCATGCAG  TACACCCGTC -301
BrHyPRP03   F -802 CCTTATTAAG  CATGCATGCATGCAG  CTGACCGACC -768
ZmHyPRP01   F -286 CACCGCCGAT  CATGCATGCACGCAG  ACACCAGCTG -252
```

Motif 3

```
OsHyPRP05 F -595 ATTGGCCCTT  GCCTCCCGCAGGTAT  ATTATTGCAT -561
BrHyPRP09 F -243 GTTCTGTGAT  GCCACCCCCTTGTCT  AGATCCTTCC -209
SbHyPRP06 F -252 GCTTCCAAAG  CCCACCCCATTGTCT  TCTTCCTCGT -218
BrHyPRP03 R -79  TCTCATGTGT  GGCTCCCGCAGGTCT  AAACGTGGAT -45
ZmHyPRP01 F -146 TTAAAACTCT  GCTTCCCCCTCCTCT  CAATCTTTTA -112
```

Supplemental FIGURE S7 Sequence around the estimated conserved motif. F and R indicate forward and reverse strands, respectively. The conserved motif is underlined.

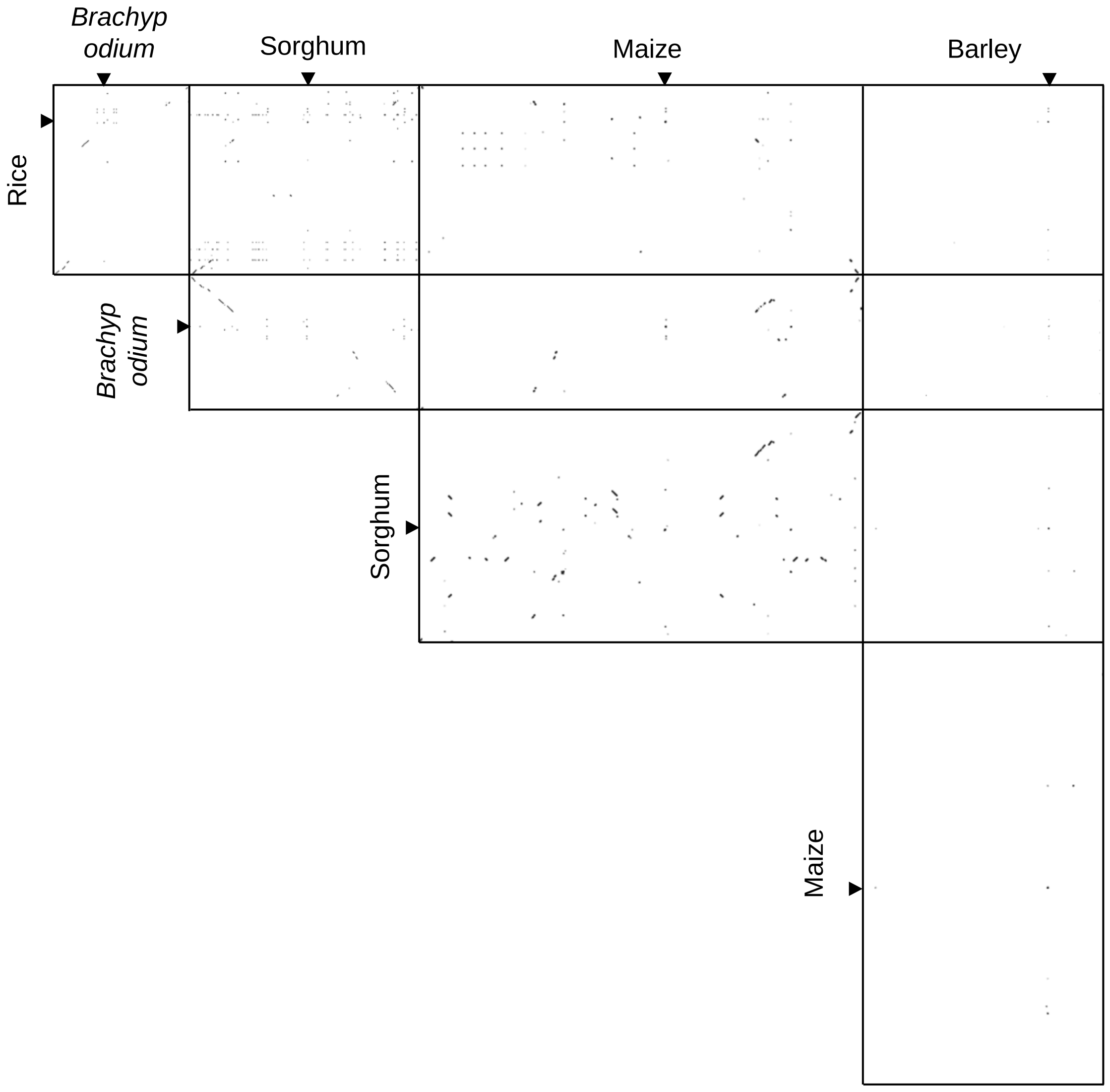

Supplemental FIGURE S8 Dot plot analysis to identify the chromosomal regions around *qLTG3-1* orthologous genes among monocots. Arrowheads indicate the position of the orthologous genes.

**Supplemental Table S1 Primer sequences for RT-PCR experiments**			
**Gene name**	**Forward**	**Reverse**	**Product size (bp)**
OsHyPRP01	TCGGAATCAACCTCAACCTC	CGTACGGATAGCGGGAATAA	338
OsHyPRP02	GCCGTCCTACAACACCAAGT	ATATGCATGCAGGCACTCAA	371
OsHyPRP03	ATGTGCTCTTCACCATGCAG	GAGATTGATGCCGAGGATGT	293
OsHyPRP04	GTGTTCGTCGTTGCTCTGAA	ATCAGATGAGTGGGCAGGAG	392
OsHyPRP05/qLTG3-1	GCCTCCTCGTCAACTACTGC	ACCGATGGATCGAACAAGAG	443
OsHyPRP06	AACCTGGTGGTGTTCTCGTT	AATAGCCAGCGGAACAAATG	401
OsHyPRP07	GTGCTGGGCCTCATCAAG	CCATGCATGACAAGTGACAA	318
OsHyPRP08	TTGATCTTCTGTCGCAAACG	TGCAAAGCACGAGAAAAATG	446
OsHyPRP09	CAATGGCTTCCAAGGTCATC	CAGCTCAGCAAGTGAAATCG	420
OsHyPRP10	GCTGAGAGTGTGCGCTAATG	AACAAACCTCGACACCCAAC	291
OsHyPRP11	TAGCCTCCTCCTCTTTGCTG	TGAAATCGGTCGGATAGGTC	359
OsHyPRP12	GGCTGATCGGTGTGAAGATT	GGTGGTCAAGGACGGTAGAA	370
OsHyPRP13	CTAGCTCGCTAGCTGCATCC	AGGTCTTGCCGCACTTGTT	438
OsHyPRP14	CCTTGAAGCTGAGGGTGTGT	CTGCGCATTTGTCCATGTAT	283
OsHyPRP15	AAGTCGTAGCTCCCCTCCTC	TTCAGAGCCAGTCATTGCAT	433
OsHyPRP16	GTGCTCAATGGGCTCGTC	CAATGTAGAATGGGCTGCAA	344
OsHyPRP17	CTCAGCCTCCTCCTCTTCG	GGAATCGGGATGAAAGATCA	448
OsHyPRP18	GTTCTAGCGGTGAGCCTCCT	TGCAGTGTCCCAATGTGATT	399
OsHyPRP19	GCTACAAGAAGCAGCCTAGCA	TTGAGGATGAGGCTGAGGTC	371
OsHyPRP20	GTGAGCCTCCTGGTGGTG	TGGGGGTAAAAACAACATGAA	449
OsHyPRP21	GCCTCATCCTCAACTTCTGC	GAGCGAACAAATACGCATCA	274
eIF1α(Os03g0177500)	CTCAAGCCTGGTATGGTGGT	AGGAACTTGGGCTCCTTCTC	368

Supplemental Table S2 Amino acid similarity among the HyP/GRP gene family in rice

	OsHyPRP01	OsHyPRP02	OsHyPRP03	OsHyPRP04	OsHyPRP05/qLTG3-1	OsHyPRP06	OsHyPRP07	OsHyPRP08	OsHyPRP09	OsHyPRP10	OsHyPRP11	OsHyPRP12	OsHyPRP13	OsHyPRP14	OsHyPRP15	OsHyPRP16	OsHyPRP17	OsHyPRP18	OsHyPRP19	OsHyPRP20	OsHyPRP21
OsHyPRP01	-																				
OsHyPRP02	0.742	-																			
OsHyPRP03	0.753	0.632	-																		
OsHyPRP04	0.716	0.600	0.879	-																	
OsHyPRP05/qLTG3-1	0.479	0.447	0.479	0.437	-																
OsHyPRP06	0.916	0.732	0.747	0.711	0.500	-															
OsHyPRP07	0.911	0.726	0.753	0.716	0.495	0.984	-														
OsHyPRP08	0.726	0.784	0.674	0.626	0.511	0.732	0.732	-													
OsHyPRP09	0.632	0.563	0.595	0.568	0.453	0.621	0.616	0.611	-												
OsHyPRP10	0.626	0.568	0.600	0.568	0.468	0.626	0.621	0.621	0.953	-											
OsHyPRP11	0.616	0.553	0.579	0.553	0.426	0.621	0.611	0.605	0.884	0.889	-										
OsHyPRP12	0.632	0.600	0.595	0.563	0.447	0.611	0.611	0.600	0.753	0.774	0.721	-									
OsHyPRP13	0.632	0.547	0.589	0.568	0.484	0.621	0.611	0.584	0.795	0.811	0.774	0.732	-								
OsHyPRP14	0.616	0.558	0.600	0.568	0.463	0.632	0.621	0.605	0.821	0.842	0.789	0.795	0.832	-							
OsHyPRP15	0.621	0.595	0.584	0.563	0.474	0.616	0.605	0.589	0.784	0.795	0.753	0.837	0.763	0.800	-						
OsHyPRP16	0.626	0.605	0.589	0.558	0.479	0.632	0.621	0.605	0.795	0.805	0.763	0.842	0.774	0.816	0.974	-					
OsHyPRP17	0.637	0.568	0.600	0.568	0.447	0.642	0.632	0.605	0.800	0.816	0.758	0.789	0.789	0.874	0.805	0.826	-				
OsHyPRP18	0.668	0.605	0.584	0.553	0.442	0.647	0.642	0.589	0.663	0.689	0.668	0.700	0.674	0.695	0.716	0.721	0.716	-			
OsHyPRP19	0.705	0.647	0.626	0.595	0.405	0.684	0.679	0.611	0.647	0.668	0.653	0.679	0.647	0.674	0.684	0.689	0.684	0.879	-		
OsHyPRP20	0.658	0.605	0.589	0.563	0.432	0.647	0.642	0.600	0.663	0.684	0.653	0.700	0.663	0.674	0.695	0.700	0.695	0.895	0.868	-	
OsHyPRP21	0.605	0.516	0.537	0.500	0.653	0.626	0.621	0.516	0.500	0.495	0.474	0.468	0.511	0.495	0.495	0.500	0.484	0.511	0.474	0.495	-

Supplemental Table S3 Identity of the 1 kb upstream regions among the HyP/GRP gene family in rice

	OsHyPRP01	OsHyPRP02	OsHyPRP03	OsHyPRP04	OsHyPRP05/qLTG3-1	OsHyPRP06	OsHyPRP07	OsHyPRP08	OsHyPRP09	OsHyPRP10	OsHyPRP11	OsHyPRP12	OsHyPRP13	OsHyPRP14	OsHyPRP15	OsHyPRP16	OsHyPRP17	OsHyPRP18	OsHyPRP19	OsHyPRP20	OsHyPRP21
OsHyPRP01																					
OsHyPRP02	0.762																				
OsHyPRP03	0.698	0.643																			
OsHyPRP04	0.705	0.642	0.909																		
OsHyPRP05/qLTG3-1	0.488	0.432	0.453	0.455																	
OsHyPRP06	0.916	0.771	0.687	0.698	0.477																
OsHyPRP07	0.893	0.776	0.684	0.689	0.470	0.962															
OsHyPRP08	0.752	0.818	0.663	0.647	0.428	0.747	0.757														
OsHyPRP09	0.692	0.652	0.654	0.636	0.409	0.678	0.671	0.661													
OsHyPRP10	0.698	0.661	0.659	0.645	0.428	0.677	0.668	0.661	0.918												
OsHyPRP11	0.678	0.631	0.629	0.619	0.406	0.654	0.643	0.642	0.865	0.862											
OsHyPRP12	0.699	0.692	0.668	0.666	0.416	0.698	0.691	0.640	0.795	0.794	0.766										
OsHyPRP13	0.656	0.633	0.652	0.640	0.420	0.628	0.626	0.636	0.734	0.748	0.719	0.727									
OsHyPRP14	0.685	0.649	0.680	0.666	0.430	0.664	0.663	0.647	0.780	0.785	0.748	0.783	0.818								
OsHyPRP15	0.708	0.699	0.675	0.677	0.434	0.705	0.698	0.673	0.792	0.795	0.773	0.837	0.740	0.809							
OsHyPRP16	0.705	0.699	0.678	0.673	0.430	0.698	0.692	0.675	0.801	0.801	0.776	0.839	0.755	0.827	0.969						
OsHyPRP17	0.699	0.670	0.698	0.687	0.428	0.685	0.684	0.680	0.797	0.804	0.776	0.799	0.801	0.885	0.853	0.872					
OsHyPRP18	0.804	0.745	0.703	0.698	0.465	0.781	0.781	0.717	0.699	0.710	0.671	0.691	0.689	0.705	0.708	0.699	0.717				
OsHyPRP19	0.748	0.713	0.687	0.680	0.446	0.752	0.760	0.668	0.677	0.671	0.645	0.715	0.647	0.692	0.720	0.717	0.719	0.857			
OsHyPRP20	0.797	0.745	0.696	0.694	0.474	0.797	0.797	0.705	0.684	0.682	0.657	0.692	0.663	0.678	0.701	0.698	0.694	0.899	0.893		
OsHyPRP21	0.570	0.502	0.505	0.507	0.629	0.561	0.558	0.502	0.476	0.490	0.469	0.484	0.476	0.495	0.500	0.497	0.497	0.531	0.495	0.516	

Supplemental Table S4 OsHyPRP21 alleles in the world rice core collection

ID	Name	Origin[a]	Cultivar group[b]	Cultivar group[c]	Allele
WRC01	Nipponbare	Japan	A	TeJ	A
WRC04	Jena 35	Nepal	B	Ind_I	A
WRC05	Naba	India	C	Ind_III	D
WRC06	Puluik Arang	Indonesia	C	Ind_III	D
WRC07	Davao 1	Philippines	C	Ind_III	C
WRC09	Rinsisan Hngmi	China	C	Ind_III	C
WRC10	Qing Zhou Zhong	China	C	Ind_III	C
WRC11	Jinguoyin	China	C	Ind_II	D
WRC13	Asu	Bhutan	C	Ind_III	D
WRC14	IR 58	Philippines	C	Ind_II	D
WRC15	Co 13	India	C	Ind_II	D
WRC17	Keiboba	China	C	Ind_III	C
WRC18	Qingyu	China	C	Ind_II	C
WRC19	Deng Pao Zhai	China	C	Ind_II	C
WRC20	Tadukan	Philippines	C	Ind_II	C
WRC21	Shwe Nang Gyi	Myanmar	C	Ind_III	D
WRC22	Calotoc	Philippines	A	TrJ_I/Ind_III	C
WRC23	Lebed	Philippines	C	TrJ_I/Ind_III	A
WRC24	Pinulupot 1	Philippines	C	TrJ_I/Ind_III	A
WRC25	Muha	India	B	Ind_I	A
WRC27	Nepal 8	Nepal	B	Ind_I	A
WRC29	Kalo Dhan	Nepal	B	Ind_I	A
WRC30	Anjana Dhan	Nepal	B	Ind_I	A
WRC31	Shoni	Bangladesh	B	Ind_I	A
WRC32	Tupa 121-3	Bangladesh	B	Ind_I	A
WRC33	Surjamukhi	India	B	Ind_I	A
WRC34	ARC 7291	India	B	Ind_I	A
WRC35	ARC 5955	India	B	Ind_I	A
WRC37	ARC 7047	India	B	Ind_I	A
WRC38	ARC 11094	India	B	Ind_I	A
WRC39	Badari Dhan	Nepal	B	Ind_I	A
WRC40	Nepal 555	India	B	Ind_I	B
WRC41	Kaluheenati	Sri Lanka	B	Ind_I	A
WRC43	Dianyu 1	China	A	Ind_II/TeJ	A
WRC44	Basilanon	Philippines	C	TrJ_I/Ind_II	C
WRC45	Ma sho	Myanmar	A	TrJ_III	A
WRC46	Khao Nok	Laos	A	mixture	A
WRC48	Khau Mac Kho	Vietnam	A	TrJ_II	A
WRC49	Padi Perak	Indonesia	A	TrJ_I	A
WRC50	Rexmont	USA	A	TrJ_I	A
WRC53	Tima	Bhutan	A	mixture	A
WRC55	Tupa729	Bangladesh	A	mixture	A
WRC57	Milyang 23	Korea	C	Ind_II	D
WRC58	Neang Menh	Cambodia	C	Ind_III	C
WRC59	Neang Phtong	Cambodia	C	Ind_III	D
WRC60	Hakphaynhay	Laos	C	Ind_III	C
WRC61	Radin Goi Sesat	Malaysia	C	Ind_III	A
WRC62	Kemasin	Malaysia	C	Ind_III	C
WRC63	Bleiyo	Thailand	C	Ind_III	D
WRC65	Rambhog	India	C	Ind_III	A
WRC66	Bingala	Myanmar	C	Ind_III	C
WRC67	Phulba	India	A	TrJ_II	A
WRC68	Khao Nam Jen	Laos	A	TrJ_II/TeJ	A
WRC97	Chin Galay	Myanmar	C	Ind_III	C
WRC98	Deejiaohualuo	China	C	Ind_II	C
WRC99	Hong Cheuh Zai	China	C	Ind_II	C
WRC100	Vandaran	Sri Lanka	C	Ind_III	C

[a] *The origin of each accession is followed by the accession name registered in the Genebank passport data of the National Institute of Agrobiological Sciences*

[b] *Classification proposed by Kojima et al. (2005)*

[c] *Classification proposed by Ebana et al. (2010)*

Supplemental Table S5 The HyP/GRP gene family in four monocots

Species	Gene ID	DB ID	Motif	Chr	Position		Amino acids
Hordeum vulgare	HvHyPRP01	MLOC_44938		1	49,851,667	49,852,340	120
	HvHyPRP02	MLOC_72235		1	263,729,210	263,730,813	146
	HvHyPRP03	MLOC_44190		2	543,239,588	543,240,556	127
	HvHyPRP04	MLOC_19132		4	4,735,161	4,736,154	137
	HvHyPRP05	MLOC_12610		4	473,305,140	473,305,967	146
	HvHyPRP06	MLOC_66801	Gly-rich	4	536,305,903	536,306,913	133
	HvHyPRP07	MLOC_33896	Gly-rich	4	539,163,220	539,164,215	193
	HvHyPRP08	MLOC_10843		4	540,770,920	540,771,716	127
	HvHyPRP09	MLOC_20972		7	25,809,644	25,810,326	127
	HvHyPRP10	MLOC_45409		7	569,060,130	569,060,693	132
	HvHyPRP11	MLOC_40798		7	569,158,759	569,159,272	132
	HvHyPRP12	MLOC_78238	Pro-rich	NA	-	-	265
Brachypodium distachyon	BrHyPRP01	Bradi1g48160	Pro-rich	1	46,753,173	46,754,289	240
	BrHyPRP02	Bradi1g78240		1	74,296,766	74,297,655	145
	BrHyPRP03	Bradi1g78250	Gly-rich	1	74,302,377	74,303,477	166
	BrHyPRP04	Bradi1g78260		1	74,311,265	74,311,908	153
	BrHyPRP05	Bradi1g78270		1	74,313,637	74,313,984	116
	BrHyPRP06	Bradi3g32910		3	35,305,499	35,305,975	159
	BrHyPRP07	Bradi3g32940		3	35,337,154	35,337,908	120
	BrHyPRP08	Bradi3g50900		3	52,034,091	52,034,462	124
	BrHyPRP09	Bradi5g17930	Gly-rich	5	21,056,071	21,056,977	191
	BrHyPRP10	Bradi5g21530	Pro-rich	5	24,127,114	24,127,996	165
Zea mays	ZmHyPRP01	GRMZM2G379898	Gly-rich	1	1,874,719	1,876,326	190
	ZmHyPRP02	GRMZM2G351505		1	88,643,336	88,643,764	143
	ZmHyPRP03	GRMZM2G037255		1	88,858,708	88,858,307	134
	ZmHyPRP04	GRMZM2G094639	Gly-rich	1	92,473,890	92,474,567	226
	ZmHyPRP05	GRMZM2G429000		1	138,835,111	138,834,710	134
	ZmHyPRP06	GRMZM2G391272		1	138,860,135	138,859,731	135
	ZmHyPRP07	GRMZM2G391286		1	138,883,976	138,883,581	132
	ZmHyPRP08	GRMZM2G091534		1	138,887,138	138,886,752	129
	ZmHyPRP09	GRMZM2G345700	Pro-rich	2	10,546,420	10,545,983	146
	ZmHyPRP10	GRMZM2G162276		2	19,824,488	19,824,087	134
	ZmHyPRP11	GRMZM2G406313		4	152,999,565	152,999,960	132
	ZmHyPRP12	GRMZM2G410338		5	192,583,651	192,583,271	127
	ZmHyPRP13	GRMZM2G398807		5	192,666,681	192,667,067	129
	ZmHyPRP14	GRMZM2G104945		6	56,013,362	56,013,763	134
	ZmHyPRP15	GRMZM2G304378	Pro-rich	9	15,981,533	15,982,438	262
	ZmHyPRP16	GRMZM2G477685		9	117,205,470	117,205,859	130
	ZmHyPRP17	GRMZM2G477697		9	117,209,970	117,210,350	127
	ZmHyPRP18	GRMZM2G027167		9	117,238,095	117,238,484	130
	ZmHyPRP19	GRMZM2G106324		9	117,402,288	117,402,677	130
	ZmHyPRP20	GRMZM2G031354	Pro-rich	10	141,387,923	141,388,408	162
Sorghum bicolor	SbHyPRP01	Sobic.001G302600	Gly-rich	1	51,368,817	51,368,158	220
	SbHyPRP02	Sobic.001G304100		1	51,629,374	51,629,748	125
	SbHyPRP03	Sobic.001G304200		1	51,644,977	51,645,543	189
	SbHyPRP04	Sobic.001G304300		1	51,651,333	51,651,800	156
	SbHyPRP05	Sobic.001G541300		1	73,245,435	73,245,887	151
	SbHyPRP06	Sobic.001G541400	Gly-rich	1	73,279,455	73,281,049	176
	SbHyPRP07	Sobic.001G542000		1	73,364,538	73,364,951	138
	SbHyPRP08	Sobic.004G291500		4	62,546,582	62,546,187	132
	SbHyPRP11	Sobic.004G292000		4	62,599,993	62,585,382	123
	SbHyPRP09	Sobic.004G292100		4	62,589,594	62,589,226	123
	SbHyPRP10	Sobic.004G292200		4	62,596,141	62,595,773	123
	SbHyPRP12	Sobic.004G292300		4	62,606,524	62,606,156	123
	SbHyPRP13	Sobic.004G292400		4	62,613,081	62,612,713	123
	SbHyPRP14	Sobic.004G292500		4	62,616,931	62,616,563	123
	SbHyPRP15	Sobic.004G292600		4	62,623,484	62,623,116	123
	SbHyPRP16	Sobic.004G292700		4	62,627,334	62,626,966	123
	SbHyPRP17	Sobic.004G292800		4	62,633,881	62,633,513	123
	SbHyPRP18	Sobic.004G292900		4	62,647,111	62,646,752	120
	SbHyPRP19	Sobic.005G046700		5	4,459,873	4,459,478	132
	SbHyPRP20	Sobic.006G172500		6	53,770,304	53,770,696	131
	SbHyPRP21	Sobic.006G172600		6	53,773,276	53,779,144	130
	SbHyPRP22	Sobic.006G172700		6	53,789,428	53,789,820	131
	SbHyPRP23	Sobic.008G158300	Gly-rich	8	51,755,343	51,764,073	190
	SbHyPRP24	Sobic.008G158600	Gly-rich	8	51,779,222	51,780,305	230
	SbHyPRP25	Sobic.008G158700	Gly-rich	8	51,797,998	51,803,156	230
	SbHyPRP26	Sobic.008G158800	Gly-rich	8	51,807,366	51,808,558	233
	SbHyPRP27	Sobic.010G003600		10	305,525	305,926	134
	SbHyPRP28	Sobic.010G054800	Pro-rich	10	4,295,822	4,294,848	325

Supplemental Table S6 Amino acid similarity among *qLTG3-1* orthologous genes in monocots

	HvHyPRP07	BrHyPRP03	BrHyPRP09	ZmHyPRP01	SbHyPRP06	SbHyPRP23	SbHyPRP24	SbHyPRP25	SbHyPRP26	OsHyPRP05
HvHyPRP07	-									
BrHyPRP03	0.846	-								
BrHyPRP09	0.786	0.722	-							
ZmHyPRP01	0.850	0.778	0.752	-						
SbHyPRP06	0.808	0.756	0.714	0.863	-					
SbHyPRP23	0.786	0.718	0.705	0.803	0.765	-				
SbHyPRP24	0.697	0.603	0.590	0.705	0.671	0.748	-			
SbHyPRP25	0.697	0.603	0.590	0.701	0.667	0.744	0.996	-		
SbHyPRP26	0.679	0.590	0.568	0.692	0.658	0.731	0.966	0.962	-	
OsHyPRP05	0.833	0.748	0.726	0.838	0.880	0.744	0.654	0.654	0.645	-

Supplemental Table S7 Identity among the upstream regions of *qLTG3-1* orthologs in monocots

	SbHyPRP06	SbHyPRP23	SbHyPRP24	SbHyPRP25	SbHyPRP26	BrHyPRP03	BrHyPRP09	ZmHyPRP01	OsHyPRP05
SbHyPRP06	-								
SbHyPRP23	0.432	-							
SbHyPRP24	0.436	0.756	-						
SbHyPRP25	0.277	0.513	0.597	-					
SbHyPRP26	0.279	0.500	0.612	0.748	-				
BrHyPRP03	0.406	0.425	0.421	0.299	0.295	-			
BrHyPRP09	0.448	0.462	0.483	0.318	0.313	0.401	-		
ZmHyPRP01	0.417	0.428	0.421	0.342	0.332	0.403	0.410	-	
OsHyPRP05	0.421	0.403	0.411	0.285	0.274	0.415	0.434	0.395	-

Supplemental Table S8 Gene list in the chromosomal regions around *qLTG3-1* orthologous genes

**Rice Chromosome 3**				**Rice Chromosome 10**				***Brachypodium* chromosome 1**				**Maize chromosome 1**				**Sorghum chromosome 1**			
**Category**	**Locus ID**	**Position**	**Description**	**Category**	**Locus ID**	**Position**	**Description**	**Category**	**Locus ID**	**Position**	**Description**	**Category**	**Locus ID**	**Position**	**Description**	**Category**	**Locus ID**	**Position**	**Description**
C	Os03g0102700	189695	expansin precursor, putative, expressed	C	Os10g0555600	21830158	Beta-expansin precursor.	C	Bradi1g78340	74657281	-	C	GRMZM2G474194	1662969	-	C	Sobic.001G542100	73375648	-
					Os10g0555300	21809273	Zinc finger, C2H2-like domain containing protein.												
					Os10g0555200	21802742	Resolvase, holliday junction-type, YqgF-like domain containing protein.												
					Os10g0555100	21798929	Similar to DNA chromosome 4, ESSA I CONTIG fragment NO. 6 (Glucosyltransferase like protein).												
					Os10g0554900	21787628	Protein of unknown function DUF566 family protein.												
					Os10g0554800	21786115	OsHyPRP21												
					Os10g0554300	21774188	Flavin monooxygenase-like domain containing protein.												
									Bradi1g78335	74655446	-								
													GRMZM2G703462	1752158	-				
																	Sobic.001G542000	73364349	HyPRP07
B	Os03g0103000	202527	Similar to Nitrate transporter	B	Os10g0554200	21757771	Nitrate transporter.	B	Bradi1g78330	74640022	-	B	GRMZM2G161483	1760349	-	B	Sobic.001G541900	73354531	similar to the Nitrate/chlorate transporter, putative, expressed
				8	Os10g0554100	21752345	Similar to Oxysterol-binding protein.	8	Bradi1g78320	74634095	-					8	Sobic.001G541800	73348789	similar to the Putative uncharacterized protein
				7	Os10g0554001	21741488	Conserved hypothetical protein.	7	Bradi1g78310	74626751	-								
				6	Os10g0553900	21731157	Protein of unknown function DUF863, plant family protein.	6	Bradi1g78300	74619298	-								
				5	Os10g0553800	21724435	Flavin-containing monooxygenase FMO family protein.	5	Bradi1g78290	74612716	-					5	Sobic.001G541700	73322908	-
				4	Os10g0553600	21721801	Exostosin-like family protein.	4	Bradi1g78280	74608540	-	4	GRMZM2G161540	1777859	-	4	Sobic.001G541600	73320124	similar to the Os10g0553600 protein
	Os03g0103100	208340	OsHyPRP03		Os10g0553300	21707054	Similar to Trehalose-6-phosphate phosphatase.		Bradi1g78270	74597313	HyPRP05		GRMZM2G461427	1785657	-		Sobic.001G541500	73316830	similar to the Os01g0332900 protein
	Os03g0103200	211082	OsHyPRP04		Os10g0552900	21700753	Crotonase, core domain-containing protein.		Bradi1g78260	74594842	HyPRP04		GRMZM2G161560	1785557	-		Sobic.001G541400	73279455	SbHyPRP06
	Os03g0103300	220396	OsHyPRP05/qLTG3-1		Os10g0552800	21697077	OsHyPRP20		Bradi1g78255	74590973	-		GRMZM2G579667	1786833	-		Sobic.001G541300	73244996	SbHyPRP05
					Os10g0552700	21693876	OsHyPRP19		Bradi1g78250	74586062	HyPRP03		GRMZM2G079891	1857691	-				
					Os10g0552600	21690332	OsHyPRP18		Bradi1g78246	74584156	-		GRMZM2G379918	1872319	-				
					Os10g0552400	21679094	Zinc finger, RING/FYVE/PHD-type domain-containing protein.		Bradi1g78240	74580402	HyPRP02		GRMZM2G379898	1874719	ZmHyPRP01				
					Os10g0552300	21674707	OsHyPRP17		Bradi1g78235	74576311	-		GRMZM2G162208	1895702	-				
					Os10g0552200	21670510	OsHyPRP16												
					Os10g0551900	21662454	OsHyPRP14												
					Os10g0551800	21658192	OsHyPRP13												
					Os10g0551700	21655489	Plant lipid transfer protein and hydrophobic protein, helical domain-containing protein.												
					Os10g0551600	21652033	Conserved hypothetical protein.												
A	Os03g0103400	235340	GRAS transcription factor domain-containing protein	A	Os10g0551200	21635191	Similar to the Scl1 protein (Fragment).	A	Bradi1g78230	74572323	-	A	GRMZM2G060265	1953455	-	A	Sobic.001G541200	73214103	-
								9	Bradi1g78220	74562938	-	9	GRMZM2G060296	1960833	-	9	Sobic.001G541100	73203331	similar to the Putative uncharacterized protein
	Os03g0103600	254279	unknown protein																
	Os03g0103700	262997	unknown protein																
	Os03g0103800	268645	unknown protein																
	Os03g0103950	272404	unknown protein																
	Os03g0104000	274345	Viral A-type inclusion protein repeat-containing protein																
	Os03g0104100	276806	Protein phosphatase 2C, N-terminal domain-containing protein																
	Os03g0104400	295403	Protein of unknown function DUF1677, Oryza sativa domain-containing protein.																
	Os03g0104800	305896	Hypothetical conserved gene.																
	Os03g0104901	309311	Conserved hypothetical protein.																
	Os03g0105000	313806	Hypothetical protein.																
	Os03g0105300	321593	Protein of unknown function DUF1677, Oryza sativa domain-containing protein.																
	Os03g0105401	324659	Hypothetical gene.																
	Os03g0105500	337912	Conserved hypothetical protein.																
					Os10g0551100	21623619	Conserved hypothetical protein.												
					Os10g0550900	21618211	Proline oxidase domain-containing protein.												
					Os10g0550800	21615390	Hypothetical conserved gene.												
					Os10g0550775	21612230	Conserved hypothetical protein.												
									Bradi1g78215	74558142	-								
													GRMZM5G827690	1969168	-				
													GRMZM2G176952	1969043	-				
																	Sobic.001G541000	73196607	similar to Terminal acidic SANT 1
1	Os03g0105600	339986	Tubulin beta-1 chain (Beta-1 tubulin).					1	Bradi1g78210	74554494	-	1	GRMZM2G164696	2035661	-	1	Sobic.001G540900	73194243	similar to Tubulin beta-2/beta-3 chain
2	Os03g0105700	344309	Protein of unknown function DUF296 domain-containing protein					2	Bradi1g78200	74550745	-					2	Sobic.001G540800	73187353	weakly similar to DNA-binding family protein, putative, expressed
3	Os03g0105800	348432	Hypothetical conserved gene.	3	Os10g0550750	21607401	Protein of unknown function DUF3527 domain-containing protein.2193	3	Bradi1g78190	74543523	-	3	GRMZM2G703466	2040712	-	3	Sobic.001G540700	73178997	similar to Os03g0105800 protein

qLTG3-1 orthologous genes are colored in yellow

Genes showing collinearity are colored in light orange
